# The influence of age on emotion-regulatory strategies, emotional work demands, and health/work outcomes: a systematic PRISMA literature review with narrative synthesis

**DOI:** 10.1186/s40359-026-05277-9

**Published:** 2026-08-05

**Authors:** Tímea Zsuzsanna Popucza, Lars Eriksson, Bernice Skytt, Anna Bjärtå, Mårten Eriksson

**Affiliations:** 1 Department of Occupational Health, Psychology and Sport Sciences, University of Gävle, Gävle, 801 76 Sweden; 2 Department of Caring Sciences, University of Gävle, Gävle, Sweden; 3 Department of Public Health and Caring Sciences, Uppsala University, Uppsala, Sweden; 4 Department of Psychology and Social Work, Mid Sweden University, Östersund, Sweden

**Keywords:** Age, Emotional work demands, Emotion regulation, Emotional labour, Health/work outcomes

## Abstract

**Background:**

An increasing age of the workforce necessitates a better understanding of age-related differences in emotions at work to inform age-inclusive workplace policies and practices. This review critically evaluates how age relates to emotion-regulatory strategies at work (emotional labour and emotion regulation), emotional work demands, and associated health and work outcomes.

**Methods:**

Following PRISMA guidelines, we systematically reviewed studies examining relationships between age, emotion-regulatory strategies, emotional work demands, and health/work outcomes. A completed PRISMA 2020 checklist is provided in the Supplementary Materials. Twenty-six studies were included and synthesized narratively.

**Results:**

Across studies, no generalisable pattern of age-related differences emerged. While several studies reported age differences in the associations between emotion-regulatory strategies and health or work outcomes, often indicating comparatively more favourable outcomes among older workers, these findings were not consistently observed across studies, with some studies reporting reversed or null effects. Moreover, studies reporting age differences varied in what aspect these differences reflected, including variation in the frequency or type of strategies used, as well as differences in how these strategies related to outcomes across age. Similarly, associations between emotional work demands and outcomes varied by age in some studies but did not follow a uniform pattern. Overall, the evidence suggests that while age-related differences in these associations are sometimes observed, they are inconsistent, not systematically associated with advantage or disadvantage for any age group, and their underlying mechanisms remain unclear.

**Conclusion:**

Age did not emerge as a robust determinant of how emotion-regulatory strategies or emotional work demands relate to health and work outcomes. The findings underscore the need for more integrative, theoretically grounded, and methodologically rigorous approaches that better account for how age, emotional work demands, regulation, and outcomes are linked, while addressing key conceptual and design challenges.

**Trial registration:**

PROSPERO CRD42022300000, published on 10 May 2022.

**Supplementary Information:**

The online version contains supplementary material available at 10.1186/s40359-026-05277-9.

## Introduction

Increasing life expectancy and better overall health make it feasible and necessary to extend working lives [1]. This shift implies both challenges and benefits. While aging is associated with declines in certain cognitive functions [2], a review of emotional competencies in work and organizational contexts found that older workers generally function similarly to, or slightly better than, younger workers across most emotional domains, including emotional control, emotion-regulation knowledge, understanding others’ emotions, and some aspects of regulating one’s own emotions. However, evidence for age-related differences in specific emotion-regulatory processes and strategies remains limited and mixed [3]. Some research further suggests that age-related differences in emotional experience after a day of work may operate indirectly through differences in the use of emotion-regulatory strategies rather than through direct age differences in emotional experience itself [4]. Such patterns may have implications for work roles involving emotion regulation at work. Some research has claimed that older workers may be more vulnerable to situations that elicit emotional arousal, due to age-related decreases in physiological flexibility [5]. However, existing research has typically examined emotional work demands, emotion-regulatory strategies, and their age-related differences in isolation, limiting understanding of how age may influence their associations with health and work outcomes. To address this gap, the present review critically examines how age is related to both emotion-regulatory strategies and emotional work demands, in relation to health and work outcomes.

### Emotion-regulatory strategies at work

Hochschild [6] described emotional labour as the effort to manage emotional expressions to meet job-related display rules through surface or deep acting. Surface acting involves displaying emotions that do not match one’s internal experience, while deep acting refers to the regulation of internal emotional experience, whereby individuals attempt to induce or reshape felt emotions so that their outward expression becomes authentic. Emotional labour is therefore typically conceptualized as regulation directed toward fulfilling external display rules, often in customer- or client interactions. In contrast, emotion regulation more broadly refers to “the processes by which individuals influence which emotions they have, when they have them, and how they experience and express” [7, p. 275] them. These processes may be automatic or controlled and may occur in response to internal goals or external situational demands. Gross’s [7] process model distinguishes between antecedent-focused strategies (e.g., reappraisal) and response-focused strategies (e.g., suppression). Grandey [8] integrated these perspectives by conceptualizing emotional labour as a specific form of emotion regulation enacted in the workplace. In this framework, deep acting corresponds to antecedent-focused regulation (e.g., reappraisal), whereas surface acting corresponds to response-focused regulation (e.g., suppression). In the present review, we include studies examining both display-rule-driven emotional labour and more general forms of emotion regulation at work, as both concern the management of emotional experience or expression in occupational contexts, albeit enacted for potentially different purposes. We use the term emotion-regulatory strategies as an umbrella term encompassing these related but conceptually distinct processes. This distinction is important for the present review, as studies differ in whether they examine regulation driven by organizational display rules or more general forms of emotion regulation, which may influence how age-related differences in emotional processes and their associations with health and work outcomes are conceptualized and interpreted. Broader coping constructs are not included as standalone categories, as they typically reflect general stress-management processes beyond emotion-regulatory strategies. However, coping strategies are included when they conceptually overlap with emotion-regulatory strategies (e.g., cognitive reappraisal).

### Emotional work demands

The original definition of emotional labour by Hochschild [6] included both the management of feelings (through surface and deep acting) and the organizationally prescribed display rules that necessitate such management. Specifically, a job-focused perspective conceptualizes emotional labour as the emotional work demands and display rules inherent in a role, whereas an employee-focused perspective defines it as the individual process of regulating emotions and emotional expressions in response to those demands [9]. A separation between emotional work demands and emotion-regulatory processes makes it possible to apply a general stress perspective on emotional work, distinguishing between demands as exposure to stressors and regulatory processes as potential mechanisms linking demands to health and work outcomes. This separation also clarifies the relevance of different occupational health and stress theories, for studying emotions at work, for example, the Job Demands-Resources model [10, 11], the Job Demands-Control-Support model [12], and the Conservation of Resources theory [13].

Within occupational health science, emotional work demands are typically framed as job characteristics, either as external requirements of the work role or as exposure to emotionally taxing work environments [9, 14]. These demands may include expectations to display or hide emotions, exposures to emotionally salient work situations, or the subjective experience of emotional strain [15–19]. Thus, emotional work demands are conceptually distinct from emotion-regulatory strategies, and some models suggest a sequential relationship, where emotional work demands trigger emotion regulation, which in turn can influence health/work outcomes [19–21]. The present review follows this practice and includes emotional work demands and emotion-regulatory strategies as distinct but related concepts and explores how they are associated with age and health/work outcomes.

Although emotional dissonance is frequently examined in the emotional labour literature, it fell outside the scope of this review due to conceptual inconsistencies. For instance, Zapf [19] and Hülsheger and Schewe [17] conceptualize emotional dissonance as a work environment exposure that contributes to strain. In contrast, Abraham [22] frames emotional dissonance as a facet of emotional labour, while Grandey [8] describes emotional dissonance as a negative outcome resulting from emotion-regulatory efforts, particularly surface acting. Given this variability, where emotional dissonance is alternately framed as an exposure, a process, or an outcome, it was excluded from the present review.

### Age and emotional work demands and emotion-regulatory strategies

Socioemotional selectivity theory (SST) is a life-span motivational framework that explains age-related changes in social preferences and emotional experience through shifts in perceived time horizons and goal priorities [23]. As perceived future time becomes more limited, emotionally meaningful goals gain salience, and individuals become more selective in their social interactions, prioritizing relationships and contexts that are emotionally meaningful. Although SST does not explicitly theorize emotion regulation processes per se, within work and organizational research, it has been drawn upon to interpret age-related differences in emotion regulation, emotional labour, and responses to emotional demands at work [5]. In these applications, emotion-regulatory processes are typically treated as implications of the broader motivational shifts described by SST, with some researchers inferring that the prioritization of emotionally meaningful goals may influence how emotions are regulated or experienced, rather than as core mechanisms specified by the theory itself. Importantly, age-related differences discussed in this literature may reflect both how emotions are regulated and differences in exposure to and appraisal of emotional demands at work. They may also reflect broader age-related changes emphasized in SST, such as increased selectivity in social interactions. While such selectivity can conceptually overlap with situation selection as described in process models of emotion regulation (e.g., Gross [7]), it is rarely operationalized as such in workplace research, and may be structurally constrained in an organisational context.

Although other theoretical perspectives may also offer explanations for age-related patterns in emotional functioning at work, much of the existing research in this area has been guided by SST. Accordingly, the present review adopts SST as the primary organizing framework. To further specify how SST-based explanations may translate into patterns of emotion regulation under varying levels of emotional demand, the review also draws on the Strength and Vulnerability Integration (SAVI) model [5]. SAVI draws on insights from SST-informed research on aging and emotion, while extending this work by incorporating both experience-based strengths in emotion regulation (derived from accumulated experience, “time lived”) and age-related physiological vulnerabilities that emerge under sustained, unavoidable, or high-intensity conditions, which are highly relevant in emotionally demanding work contexts. From this perspective, older workers may be better equipped to manage everyday emotional work demands, yet potentially more vulnerable when emotional work demands or regulatory requirements are intense, chronic, or difficult to avoid. This distinction is particularly relevant, as emotional work demands often involve prolonged exposure to emotionally challenging situations that cannot easily be avoided or selectively managed.

While SAVI explicitly specifies how age-related differences in emotion regulation unfold, highlighting both experience-based strengths and physiological constraints, SST primarily focuses on shifts in time perspective and goal prioritization and does not explicitly theorize emotion-regulatory processes. Additionally, although numerous reviews have examined emotion regulation across the life span (e.g [3, 24]), or emotional labour and associated health and work outcomes (e.g., [17, 25, 26]), these bodies of work have largely evolved in parallel. The existing studies also differ in how these relationships are conceptualized, with some focusing on age-related differences in strategy use, others on differences in the consequences of strategy use, and others on age-related variation in responses to emotional work demands. As a result, it remains unclear whether and how age is related to emotion-regulatory strategies and emotional demands at work in shaping health and work outcomes, particularly how age-related differences in emotion regulation and exposure to emotional work demands jointly relate to such outcomes.

### Health/work outcomes

From a stress-theoretical perspective, such as the Job Demands-Resources model [11] and Conservation of Resources theory [13], emotional work demands are conceptualized as job stressors that require sustained emotional effort and may deplete energetic and psychological resources. Resource depletion may in turn impair well-being and undermine work motivation and performance, thereby linking emotional work demands to both health and work outcomes.

Consistent with this perspective, both emotional work demands and emotion-regulatory strategies have been examined in relation to a broad range of health and work outcomes. Reviews and meta-analyses on emotional labour document associations with burnout, emotional exhaustion, psychological strain, depressive symptoms, job satisfaction, performance, and turnover intentions (e.g [17, 19, 25–27]), . Complementary research on emotional work demands, grounded in job stress frameworks, similarly links emotionally demanding work characteristics to strain, impaired well-being, and adverse work outcomes such as burnout, exhaustion, and reduced job satisfaction (e.g [16, 19]), .

### Aim

**Fig. 1:**
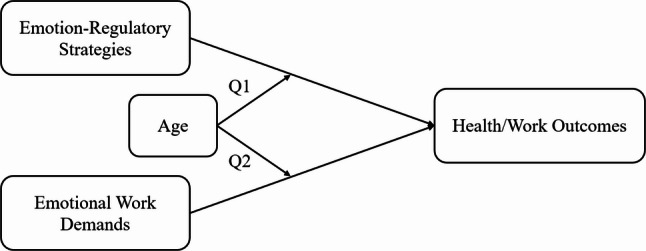
Conceptual overview of the relationships examined in the review

The present review critically examines and synthesizes how age influences the associations between emotion-regulatory strategies and health/work outcomes, and between emotional work demands and health/work outcomes. Figure 1 provides a conceptual overview of the key constructs and relationships addressed in the reviewed literature.

The review is guided by two questions:

- (Q1) How does age influence the relationship between emotion-regulatory strategies and health/work outcomes?
- (Q2) How does age influence the relationship between emotional work demands and health/work outcomes?

## Method

We conducted a systematic literature review following Preferred Reporting Items for Systematic Reviews and Meta-Analyses (PRISMA) guidelines [28], using a narrative synthesis approach based on Popay [29]. The review protocol registration was published on PROSPERO (CRD42022300000) on 10 May 2022.

### Search strategy

The search strategy was developed in collaboration with an information specialist to align with the aim and scope of the review. It included age-related terms (e.g., *age*, *elder*, *old*, *age difference*, *lifespan*, *senior*, *young*, *mature*), emotional work terms (e.g., *emotional labour*, *emotion regulation*, *emotional work demands*), and health outcomes (e.g., *burnout*, *fatigue*, *stress*, *depression*, *anxiety*, *mental health*, *recovery*, *well-being*, *vigour*), work outcomes (e.g., *job satisfaction*, *turnover*, *absenteeism*, *productivity*, *work ability*). Emotional work demands and emotion-regulatory strategies were defined at the construct level, with specific strategies (e.g., surface acting, deep acting, reappraisal, suppression) reflecting study operationalizations rather than predefined inclusion criteria. Health outcomes were defined as direct indicators of physical or mental health. General affective states not explicitly operationalized as health indicators, as well as distal labour-market outcomes (e.g., early retirement or disability pension), were not considered health outcomes for this review. Full search strings with Boolean logic and operators are provided in the Supplementary Materials.

**Fig. 2:**
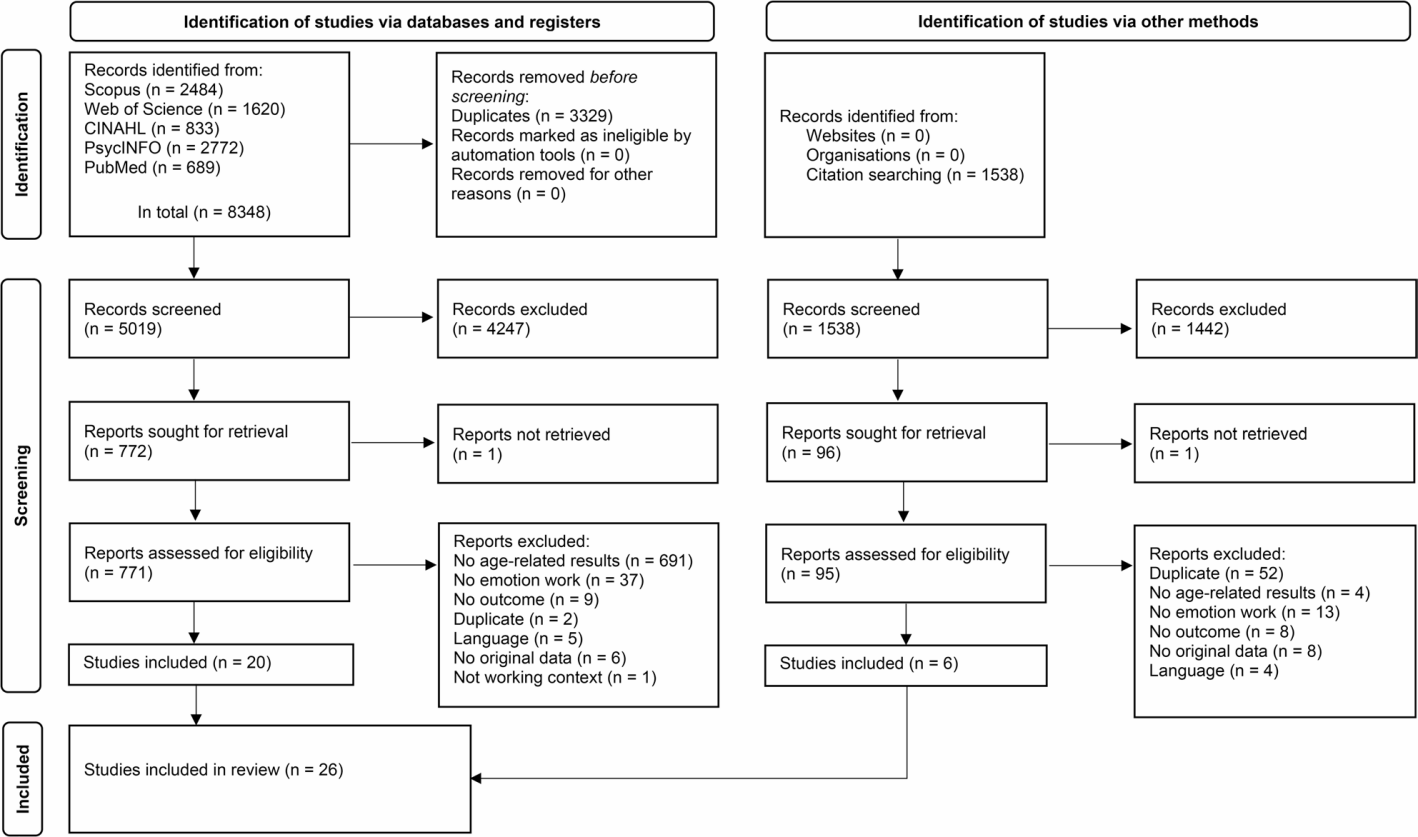
PRISMA flow chart for identification and selection of studies

Primary database searches were conducted on 5 May 2022 in Scopus, Web of Science, CINAHL, PsycINFO, and PubMed, using fields such as title, abstract, keywords, and index terms, depending on database availability. Searches were limited to peer-reviewed journal articles published in English, Swedish, Norwegian, or Danish. Citation searches (backward and forward) were performed in Scopus by the first author and screened separately. Database searches were repeated on 23 September 2023 prior to the final synthesis. Study identification and inclusion are summarised in Fig. 2 (PRISMA flow diagram; [28]).

### Eligibility criteria

We included quantitative studies based on original data that: (1) examined at least one emotional work demand, emotional labour, or emotion regulation construct or an established operationalization of these constructs (e.g., surface/deep acting, reappraisal, suppression) as a predictor, moderator, or mediator; (2) assessed its relationship to at least one health or work outcome; and (3) included at least one age-related analysis linking emotional work demand, emotional labour, or emotion regulation construct to health and/or work outcomes. Studies examining coping as a standalone construct were not eligible unless coping was operationalized as an emotion-regulatory strategy targeting emotional experience or expression at work. Studies that did not examine emotional work variables as separate predictors in relation to health or work outcomes (e.g., studies using profile analyses or latent class analyses) were excluded. The full set of screening questions used to assess eligibility is provided in Table 1.

**Table 1:** Screening questions and the response that led to an exclusion

	Level 1a – title and abstract	Exclude if
1	Does not include variables of emotional work demands, emotional labour, emotion regulation construct, or an established operationalization of these constructs at work as a predictor, mediator, or moderator.	Yes
2	Was not conducted either within work settings, regarding paid employment or on the working population.	Yes
3	Does not include at least one health or work outcome variable.	Yes
4	Is not peer-reviewed, is not fully published, was not using quantitative data, does not report a test of significance, is an article review, is a book chapter, is a book review, is a literature review, is a meta-analysis, is a theoretical or conceptual paper, is an editorial, is a commentary, is less than two pages long, was using a qualitative methodology.	Yes
5	Was not available in English, Danish, Norwegian, or Swedish.	Yes

	**Level 1b – full-text screening**	**Exclude if**
6	Should have been excluded based on the 1–5 criteria.	Yes
7	Does not present any age-related analysis and results in relation to emotional work demands, emotional labour, emotion regulation construct, or an established operationalization of these constructs and health/work outcomes.	Yes
8	Full text is unavailable.	Yes

	**Level 2 – full-text assessment**	**Exclude if**
9	Does not explicitly investigate the influence of age on the relationship between emotional work demands, emotional labour, emotion regulation construct, or an established operationalization of these constructs and health and/or work outcome(s).	Yes
10	Does not explicitly investigate how emotional work demands, emotional labour, emotion regulation construct, or an established operationalization of these constructs influence the relationship between age and work outcome(s).	Yes

### Assessment of relevance

The initial dataset (8,348 items) was imported into EndNote and deduplicated by an information specialist using the seven-step process by Bramer [30]. The remaining 5,019 items were transferred to Rayyan [31], where two reviewers independently and blindly screened titles and abstracts using Level 1a screening criteria (see Table 1).

Studies marked for inclusion or uncertainty were retrieved in full text and screened independently by two reviewers using Level 1b criteria (Table 1). Conflicts were categorized as major (inclusion vs. exclusion) or minor (inclusion/exclusion vs. uncertain). Major conflicts were resolved through full-text review by a third reviewer. Minor conflicts were resolved through abstract-level review. In cases of continued uncertainty, final decisions were made through team discussion and consensus.

Citation searches were uploaded into Zotero (v6.0.26; [32]) and deduplicated separately for backward and forward references. After deduplication, 1,538 items were uploaded to Rayyan. These were screened at the title and abstract level by the first author (Level 1a), with included and uncertain studies assessed in full text by two independent reviewers (Level 1b and Level 2). All final inclusion decisions were confirmed through team agreement.

### Rationale for exclusions

Debatable articles were discussed within the review team, and several studies were excluded after consensus. For example, Bal and Smit [33] was excluded because it assessed general affect (an emotional state) over a three-month period, rather than specific health or work outcomes. Andersen [34] was excluded as its outcome measure (risk of receiving a disability pension) did not directly reflect workers’ health or work outcomes. El Khawli [35] was excluded because emotional work variables were combined into profiles using a person-oriented analytical approach [36], rather than analyzed as separate predictors of health or work outcomes. The person-oriented approach treats patterns of variables within individuals as the primary unit of analysis, rather than estimating independent effects of specific constructs.

### Data extraction

Data extraction was performed independently by author pairs, who read the remaining articles in full and recorded key study characteristics. Extracted data included bibliographic details, study design, participant characteristics, measures of emotional work, outcome variables, relevant results, and reviewer notes. After independent reviews, notes were compared, disagreements were resolved through discussion, and final exclusion decisions were made. Next, two researchers (TZP and ME) independently extracted and diagrammed the structural models from each included study, mapping predictors, outcomes, mediators, moderators, interactions, and age-related comparisons. Diagrams were then reviewed, discrepancies discussed, and simplified to include only analyses relevant to the review questions. For each included study, we extracted all reported analyses examining associations between emotional work variables, age, and health and work outcome domains. When multiple models or time points were reported, all age-related effects of these outcome domains were recorded. No selection was made based on statistical significance.

### Quality assessment

To assess methodological quality, we reviewed available tools and selected the Quality Assessment with Diverse Studies (QuADS) tool [37], suitable for a range of study designs. QuADS includes 13 items to be rated on a four-point scale (0–3), yielding total scores from 0 to 39, with higher scores indicating stronger methodological quality. The detailed scoring rubric for the QuADS is provided in Supplementary Materials. The tool does not define cut-offs for low, moderate, or high quality. Two researchers (TZP and ME) independently rated each study, then compared scores and reached consensus through discussion. Studies were not planned to be excluded based on low quality; rather, quality appraisal scores were assessed to inform the interpretation of study limitations in the synthesis.

### Analysis strategy

Due to significant heterogeneity in study designs, measures, populations, and analytic approaches, we applied relevant steps from Popay et al.’s [29] narrative synthesis framework, which is suited to synthesizing diverse forms of evidence. Guided by established frameworks on emotional work demands and emotion-regulatory strategies, which informed our research questions and inclusion criteria, we tabulated key study characteristics and developed preliminary descriptions of the included studies. Using visualized and extracted tested assumptions (predictors, outcomes, age comparisons, mediators, and moderators), we organized studies by emotional work measures and the analytic strategies used to examine these relationships. Results were then synthesized within these groupings. Finally, we assessed the robustness of the synthesis by reflecting on study quality, theoretical alignment, conceptual definitions of emotional work and age, contextual and methodological variation, and the limitations of our review process.

Given the heterogeneity in study designs, operationalizations, outcome measures, and analytical approaches, a quantitative meta-analysis was not appropriate. Findings were therefore synthesized narratively, drawing on reported regression coefficients, interaction effects, mediation analyses, and patterns of statistical significance. Due to the absence of quantitative pooling and the diversity of designs, no formal assessment of reporting bias (e.g., funnel plots) or standardized certainty-of-evidence framework (e.g., GRADE) was applied.

### Protocol registration and amendments

During manuscript preparation, certain elements were operationalized more explicitly than described in the PROSPERO registration to enhance transparency and consistency. Specifically, inclusion criteria were clarified to state that emotional work variables had to be examined as separate predictors, mediators, or moderators in relation to health or work outcomes. This refinement did not alter the review’s scope or objectives but clarified analytic requirements. Although the PROSPERO registration indicated that different critical appraisal tools would be used, the QuADS instrument was later selected due to the heterogeneity of study designs and analytical approaches across included studies. In addition, conceptual distinctions between emotional work demands and emotion-regulatory strategies were clarified during manuscript preparation to enhance theoretical precision. These clarifications did not change the population, core constructs, or aims of the review. A meta-analysis was not conducted due to substantial heterogeneity in study designs, measures, and analytic approaches.

## Results

A total of 26 studies met the inclusion criteria. The studies were published between 2007 and 2023 and conducted primarily in European countries (e.g. the Netherlands, Germany, Poland, Croatia, Macedonia, Finland, Greece, Denmark, and Sweden), but also in China, the United States, Taiwan, India, and Thailand. Most study populations were from caring and service professions, with participant ages ranging from 16 to 79 years. Fifteen studies employed cross-sectional designs, one used a repeated cross-sectional design, six used daily experience sampling methods, and four were longitudinal. Key study characteristics are summarised in Table 2, grouped by the underlying assumptions tested.

**Table 2:** Characteristics of the included studies

Reference	Population	Age	Emotion-Regulatory Strategies/Emotional Work Demands	Outcomes	Theory	Extracted Results	Design
Emotion-regulatory strategies							
* Age as a moderator*							
Khetjenkarn and Agmapisarn [38]	Frontline hotel employees *(N =* 509)	Age range (*M*,* SD*) not reported. Participants were in their 20s to50s. Age groups of younger (*N =* 319) and older (*N =* 190) participants.	Emotional Labour Strategies assessing Surface Acting (8 items) and Deep Acting (5 items) in methods.	Burnout (22 items) on the dimensions of Emotional Exhaustion, Depersonalization, and Personal Accomplishment; Job Satisfaction (20 items); Turnover Intention (4 items), Organisational Commitment (24 items), Managers’ Emotional Intelligence (16 items)	SST	Age moderation effect. Older employees using more deep acting experience less burnout than younger ones.	Cross-sectional
Peng et al. [39]	Sample 1: Bank employees (*N =* 340) Sample 2: Employees from various occupations (*N =* 280)	25–55 years Sample 1: *M =* 40.61, *SD =* 7.79 Sample 2: *M =* 40.01, *SD =* 11.18	Emotion Regulation on the dimensions of Emotional Suppression (4 items) and Cognitive Reappraisal (6 items)	Physical Strain (12 items); Job-Related Affective Well-Being (20 emotions)	SST	Partial age moderation effects. Emotional suppression was associated with less physical strain in older workers in both samples. Suppression was also associated with higher affective well-being in older (but not younger) employees in Sample 1. Cognitive reappraisal was associated with higher affective well-being only in older workers (Sample 2).	Repeated cross-sectional with a 1-month lag between predictors (Time 1) and outcomes (Time 2).
Peng et al. [40]	Occupational and physical therapists (*N =* 614)	20–73 years old (*M =* 39.75, *SD =* 11.28)	Cognitive Reappraisal (6 items)	Workplace Deviance on the dimensions of Organisational Deviance (12 items) and Interpersonal Deviance (7 items)	SST	Age moderation effect. Older employees under abusive supervision used more cognitive reappraisal, which in turn was linked to lower organisational deviance.	Cross-sectional
Rudolph and McGonagle [41]	Full-time workers with chronic pain with jobs including customer interactions (*N =* 87)	18–63 years old (*M =* 37.83, *SD =* 9.92).	Mid- and End of Day Emotional Labour on the dimensions of Surface Acting (3 items) and Deep Acting (3 items)	Mid- and End-of-Day Perceived Work Ability (1 item)	SST	Age moderation effect. Older workers using more deep acting reported higher levels of work ability than younger ones.	Daily diary using experience sampling (5 consecutive working days)
Scheibe and Moghimi [42]	Employees (*N =* 199)	18–62 years old (*M =* 36.35, *SD =* 13.65).	Emotion Regulation Strategies of Reappraisal (3 items), Distraction (3 items), Suppression (3 items) Emotion Acceptance (3 items)	Daily Job Satisfaction (1 item), Daily Fatigue on the dimensions of Physical (1 item) Cognitive (1 item), and Emotional Fatigue (1 item)	SST	Partial age moderation effect. In response to more intense negative events, younger employees used more suppression and less acceptance, which were in turn associated with lower job satisfaction and higher fatigue.	Daily diary using experience sampling (10 days to 3 weeks)
Scheibe [43]	Employees (*N =* 123) across diverse sectors (health and social welfare, education, industry etc.)	22–63 years old (*M =* 40.8, *SD =* 13.8).	Negative Work Events (open-ended), Emotion Regulation Goal Activation (3 items); Emotion Regulation on the dimension of Expressive Suppression (3 items), Reappraisal (3 items), and Distraction (3 items)	End-of-Day Negative Affect including Negative Affect of High Arousal (4 items) and Positive Affect (12 items); Daily Attentional Focus (5 items); Daily Persistence (3 items)	SAVI	No age moderation effect in emotion regulation strategy use. Age moderation was found for emotion-regulation goal activation in response to very negative work events. Lower activation of emotion-regulation goals was associated with less end-of-day negative affect for older (compared to younger) employees.	Daily diary using experience sampling (5 days)
Walsh and Bartikowski [44]	Service employees (*N =* 237)	Age range not reported. (*M =* 31.7, *SD =* 11.2). Age groups of younger (below 28 years of age) and older (28–50 years of age) participants.	Emotional Labour on the dimensions of Surface Acting (3 items) and Deep Acting (3 items)	Job Satisfaction (3 items); Intentions to Quit (3 items)		Age moderation effect. Deep acting was positively related to job satisfaction among younger employees but not older ones. In contrast, surface acting was associated with higher quitting intentions in older, but not in younger, employees.	Cross-sectional
Yeung and Fung [45]	Insurance workers (*N =* 87)	18–61 years old (*M =* 38.55, *SD =* 11.18)	Momentary Suppression (4 items)	Job Performance assessed as Self-Rated Momentary Performance (1 item) and as Sales Productivity Statistics	SST	Age moderation effect. Greater use of suppression was associated with reduced negative affect and higher sales productivity among older, but not younger, workers.	Event-based experience sampling (5 consecutive working days)
Yeung and Wong [46]	Managerial employees (*N =* 141)	25–64 years old (*M* = 42.4, *SD* = 9.16)	Emotion Regulation on the dimensions of Emotional Suppression (4 items) and Cognitive Reappraisal (6 items)	Negative Work Events (out of 9 options, 1 item’s rating was used); Positive (4 items) and Negative (4 items) Emotions at Work, Perceived Stress (10 items); Job Satisfaction (1 item), Relationship Quality (number of items not reported)	SST	Partial age moderation effect. Older workers who habitually used more suppression reported fewer negative emotions, lower work stress, and greater job satisfaction compared to younger workers.	Daily diary using experience sampling (15 consecutive workdays)
*Age group comparisons*							
Kafetsios and Loumakou [47]	Teachers from secondary education (*N =* 475)	25–60 years old (*M =* 38.8, *SD =* 9.02). Age groups of younger (25–40, *N =* 263) and older (41–60, *N =* 212) participants.	Emotion Regulation Questionnaire (10 items) assessing Reappraisal and Emotional Suppression	Job Satisfaction (12 items), Job Affect (17 items)	SST	Age group differences. Emotion regulation strategies (reappraisal and suppression) were significant predictors of job satisfaction and affect at work in younger, but not older, employees.	Cross-sectional survey
Kaur and Malodia [48]	Doctors, nurses and paramedical staff (*N* = 586)	Age range (*M*, *SD*) not fully reported. Age groups of 25–30, 31–35, 36–40, 41–45, and 45+.	Emotional Labour (13 items) on the dimensions of Surface Acting, Deep Acting, Emotional Consonance, and Suppression	Job Satisfaction (60 items) on the dimensions of Physical, Working Conditions, Social Status, Administration, and Monetary Benefits.		No age group differences. Levels of emotional labour and job satisfaction did not differ across age groups. No further age-based analyses were reported.	Cross-sectional survey
*Emotion-regulatory strategies as mediators*							
Cheung and Tang [49]	Employees (*N =* 386) of call centre, retail, administration, and registered nurses	19–62 years old (*M* = 33.19, *SD =* 9.47).	Emotional Labour Strategies of Surface Acting (7 items), Deep Acting (4 items), and Expressing Naturally Felt Emotions (3 items)	Job Satisfaction (6 items); Psychological Distress (12 items)	SST	Support for partial mediation. Deep acting partially mediated the relationship between age and job satisfaction, and this mediation was moderated by gender.	Cross-sectional survey
Hertel et al. [50]	Workers from various occupational fields (*N =* 676 in methods).	17–73 years old (*M* = 43.47, *SD =* 10.29).	Positive Reinterpretation Scale (4 items, assessing active Emotion-Focused Coping)	Strain (6 items)	Lifespan theory of control	No support for mediation. Positive reinterpretation did not mediate the relationship between age and strain.	Longitudinal survey with 2 measurement points, 6 months apart
Johnson et al. [51]	Service employees (*N =* 444)	16–70 years old (*M =* 40.64, *SD =* 12.30).	Emotional Labour Strategies on the dimensions of Surface Acting (6 items), Anticipated Deep Acting (4 items), Situational Deep Acting (5 items), and Automatic Emotion Regulation (2 items)	Burnout dimensions of Exhaustion (5 items), Cynicism (5 items), Professional Efficacy (6 items); Work Engagement (9 items)	SST	Support for mediation. Surface acting was found to mediate the negative relationship between age and both exhaustion and cynicism. Anticipative deep acting mediated the positive relationship between age and both professional efficacy and work engagement.	Cross-sectional survey
Lee [52]	Public-service employees (*N =* 167)	25–65 years old (*M*, *SD* not reported). Groups of younger (22–44) and older (45–65) participants.	Performance of Emotional Labour (6 items), and False Face Acting (6 items)	Job Pride (7 items)		Support for mediation. Both emotional labour performance and false face acting mediated the positive relationship between age and job pride.	Cross-sectional survey
Liang et al. [53]	Nurses (*N* = 414)	Age range not specified numerically; participants divided into two groups: below and above 35 years of age.	Emotional Labour on the dimensions of Expressing Positive Emotions (5 items), Suppressing Negative Emotions (10 items), and Handling Others’ Negative Emotions (11 items)	Intent to Stay (4 items)		No support for mediation. Age was not significantly related to emotional labour, and therefore mediation models explaining intent to stay were not tested.	Cross-sectional survey
Peng et al. [54]	Full-time employees in various organisations (*N =* 158)	20–58 years old *(M =* 33.07, *SD =* 9.24).	Emotional Labour on the dimensions of Surface Acting (3 items) and Deep Acting (3 items).	Organisational Cynicism (14 items); Workplace Deviance (19 items).	SST	Support for mediation. Both surface acting and deep acting mediated the negative relationship between age and organisational cynicism and were indirectly linked to reduced workplace deviance.	Cross-sectional survey
Scheibe et al. [4]	Healthcare professionals *(N= 92)*,	17–64 years old (*M =* 43.47, *SD =* 11.14).	Emotion Regulation Strategies as Adaptive (4 strategies) and Maladaptive strategies (4 strategies)	Daily After-Work Emotions on the dimensions of High-Arousal Positive (3 emotions), Low-Arousal Positive (3 emotions), High-Arousal Negative (3 emotions), and Low-Arousal Negative (3 emotions)	SAVI	Support for partial mediation. No direct age effect was found on after-work emotions. However, age was associated with more use of adaptive and less use of maladaptive strategies, which partially explained age-related improvements in after-work emotions.	Experience sampling (1–15 days)
Sliter et al. [55]	Employees from the service industry (*N* = 519)	18–68 years old (*M =* 32.31, *SD =* 13.72).	Emotional Labour Strategies on the dimensions of Surface Acting (7 items) and Deep Acting (4 items)	Employee Well-Being was assessed as levels of Burnout on the dimensions of Physical (7 items), Emotional (7 items), and Mental Exhaustion (7 items).	SST	No support for mediation. Emotional labour strategies did not mediate the relationship between age and well-being through emotional intelligence.	Cross-sectional survey
*Emotion-regulatory strategies moderators*							
Johnson et al. [51]	Service Employees (*N =* 444)	16–70 years old (*M =* 40.64, *SD =* 12.30).	Emotional Labour Strategies on the dimensions of Surface Acting (6 items), Anticipated Deep Acting (4 items), Situational Deep Acting (5 items), and Automatic Emotion Regulation (2 items)	Burnout dimensions of Exhaustion (5 items), Cynicism (5 items), Professional Efficacy (6 items); Work Engagement (9 items)	SST	Partial support for moderation in three-way interactions. Older employees who experienced high levels of customer stressors and used more deep acting reported higher professional efficacy than younger employees under the same conditions. However, when customer stressors were low, older individuals who used less deep acting reported higher professional efficacy compared to older individuals who used more deep acting.	Cross-sectional survey
Emotional work demands							
* Age as a moderator*							
Mijakoski et al. [56]	Nurses from Croatia (*N =* 138) and Macedonia (*N =* 185)	Age range (*M*,* SD*) not reported. Croatian (*M =* 42.16, *SD =* 10.84) and Macedonian (*M =* 37.55, *SD =* 9.0) nurses.	Emotional Demands (6 items)	Burnout on the dimensions of Emotional Exhaustion (9 items) and Depersonalization (5)		Partial moderation effect. Older Macedonian (but not Croatian) nurses with high levels of emotional work demands reported higher levels of depersonalization compared to younger ones.	Cross-sectional survey
Reh et al. [57]	Workers from various professions (*N =* 2478)	20–79 years old (*M =* 47.83 *SD =* 7.95)	Emotional Job Demands (assessed as five different work activities and work characteristics)	Emotional Well-Being as Positive Affect (1 item) and Negative Affect (3 items); Job Satisfaction (1 item)	SAVI	Positive affect decreased with age and job satisfaction remained relatively stable; very high emotional job demands were associated with steeper age-related declines in both. No moderation was found for negative affect.	Accelerated longitudinal design
Scheibe et al. [58]	Senior care home employees (*N =* 141)	17–62 years old (*M =* 42.6, *SD =* 11.65)	Emotional Job Demands on the dimensions of Positive (3 items), Negative (4 items), and Neutral (3 items) Display Demands, and Emotional Sensitivity Demands (3 items)	Job Satisfaction (1 item); Need for Recovery (11 items)	SST	Partial support for age moderation. Emotional sensitivity demands were positively associated with need for recovery in older, but not in younger, employees, whereas no age-related differences were observed for job satisfaction or for positive, negative, and neutral display demands.	Cross-sectional survey
Van der Heijden et al. [59]	Nurses in the Netherlands (*N* = 3 399), and Nurses in Poland (*N* = 3 636)	Netherlands: 18–64 years (M = 38.2, SD = 9.73); Poland: 18–70 (M = 38.72, SD = 7.63) Age groups of younger (< 40 years) and older (≥ 40 years) nurses.	Emotional Demands (4 items)	Burnout (5 items), Disability (4 items), Job Satisfaction (4 items), Institutional Commitment (4 items)	SST	Partial age moderation effect. Emotional demands were associated with burnout in older (but not younger) Dutch nurses, and younger (but not older) Polish nurses.	Two-wave panel survey with waves one year apart
* Age group comparisons*							
Irehill et al. [60]	Leaders from the private sector (*N =* 1033)	18–65 years old. Age groups of younger (*M =* 27.7, *SD =* 1.5) and older (*M =* 49.6, *SD =* 8.2) participants.	Emotional Demands (4 items)	Burnout (4 items) Vigour (3 items)	Lifespan perspectives on leader roles.	No age group differences. The effects of emotional demands on burnout and vigour did not differ in younger and older leaders.	Two-wave survey, 6 months apart
Mauno et al. [61]	Nurses and physicians (*N =* 4311)	Age range (*M*,* SD*) not reported. Age groups of 18–34 years, 35–44 years, 45–54 years, and 55–65 years of age.	Emotional Sensitivity Requirements (3 items).	Recovery (1 item), Job Satisfaction (1), Dedication (3 items), and Organisational Citizenship Behaviour (number of items not reported)	SST	Age group differences. Emotional sensitivity requirements were more negatively associated with job satisfaction and work dedication among the two middle-aged groups (35–44 and 45–54 years) than among the youngest and oldest groups.	Cross-sectional survey
Zoer et al. [62]	Railway employees (*N =* 827)	22–66 years old (*M*,* SD* not reported). Age groups of 22–35 (*N =* 69); 36–45 (*N =* 235), 46–55 (*N =* 383), and 56–66 (*N =* 140).	Emotional Load (7 items) on the dimensions of low, middle, or worse	Mental Health Complaints, assessed as Burnout on the dimensions of Emotional Exhaustion (5 items), Depersonalization (5 items); Stress (16 items); Need for Recovery (11 items)		Age group differences. In the youngest group, high emotional workload was linked to increased burnout. In middle-aged groups, moderate to high emotional workload was linked to increased stress and fatigue. In the oldest group, emotional workload was not related to stress, fatigue, or burnout.	Cross-sectional survey

### Quality appraisal

Total scores on the QuADS [37] ranged from 17 to 28 (M = 23.96, SD = 2.83), as shown in Table 3. Nearly all studies scored 0 on item 12 (Evidence that the research stakeholders have been considered in research design or conduct). Other common limitations included justification for choice of study population (item 5) and rationale for the selection of data collection tools (item 6).

**Table 3:** QuADS quality appraisal scores

Reference	Sum	1.	2.	3.	4.	5.	6.	7.	8.	9.	10.	11.	12.	13.
Cheung and Tang [49]	22	3	3	0	2	1	0	3	2	2	1	2	0	3
Hertel et al. [50]	24	3	3	0	3	1	2	3	1	2	1	2	0	3
Irehill et al. [60]	28	3	3	3	3	2	0	2	1	3	2	3	0	3
Johnson et al. [51]	23	3	3	1	3	1	0	2	1	2	2	3	0	2
Kafetsios and Loumakou [47]	18	3	3	1	3	1	0	2	1	0	1	2	0	1
Kaur and Malodia [48]	17	1	3	2	3	1	0	2	0	0	1	3	0	1
Khetjenkarn and Agmapisarn [38]	21	3	3	2	3	1	0	2	1	0	1	3	0	2
Lee [52]	21	3	3	1	3	1	0	2	1	1	1	3	1	1
Liang et al. [53]	27	3	3	3	2	3	2	3	1	1	2	3	0	1
Mauno et al. [61]	24	2	3	2	3	1	1	2	2	2	1	3	0	2
Mijakoski et al. [56]	23	2	3	2	3	1	0	2	3	2	0	3	0	2
Peng et al. [54]	27	3	3	2	3	1	0	3	2	1	3	3	0	3
Peng et al. [39]	27	3	3	2	3	2	1	2	2	2	1	3	0	3
Peng et al. [40]	26	3	3	1	3	2	0	3	2	2	1	3	0	3
Reh et al. [57]	27	3	3	2	3	1	1	1	3	1	3	3	0	3
Rudolph and McGonagle [41]	27	3	3	2	2	1	1	2	3	3	1	3	0	3
Scheibe [43]	27	3	3	1	3	1	1	2	2	2	3	3	0	3
Scheibe and Moghimi [42]	25	3	3	1	3	2	0	2	2	1	2	3	0	3
Scheibe et al. [4]	24	3	3	1	3	1	0	3	2	1	1	3	0	3
Scheibe et al. [58]	25	3	3	1	3	1	1	2	2	1	2	3	0	3
Sliter et al. [55]	23	3	3	1	3	2	0	3	1	1	1	3	0	2
Van der Heijden et al. [59]	26	3	3	3	2	2	1	2	2	2	1	2	0	3
Walsh and Bartikowski [44]	22	3	3	2	2	1	1	2	1	1	1	3	0	2
Yeung and Fung [45]	27	3	3	2	3	2	1	2	2	1	2	3	0	3
Yeung and Wong [46]	24	3	3	1	3	3	2	2	1	0	1	3	0	2
Zoer et al. [62]	23	1	2	3	2	2	1	3	2	2	2	2	0	1

(1) Theoretical or conceptual underpinning to the research; (2) Statement of research aim/s; (3) Clear description of research setting and target population; (4) The study design is appropriate to address the stated research aim/s; (5) Appropriate sampling to address the research aim/s; (6) Rationale for choice of data collection tool/s; (7) The format and content of data collection tool is appropriate to address the stated research aim/s; (8) Description of data collection procedure; (9) Recruitment data provided; (10) Justification for analytic method selected; 11. The method of analysis was appropriate to answer the research aim/s; 12. Evidence that the research stakeholders have been considered in research design or conduct; 13. Strengths and limitations critically discussed

### Overview of the results

Results (see Table 2) are organized into two main sections: (1) emotion-regulatory strategies and (2) emotional work demands. In total, 19 studies examined emotion-regulatory strategies (e.g., emotional labour or emotion regulation), and seven studies examined emotional work demands (e.g., emotional demands, emotional load, sensitivity demands). All included studies examined age in relation to at least one emotional work variable and at least one health or work outcome. However, studies differed in how these associations were conceptualized and analysed. Some studies examined whether the associations between specific emotion-regulatory strategies and outcomes differed by age (i.e., whether strategies were more or less strongly related to outcomes across age), either by modelling age as a moderator or by comparing age groups. Other studies examined whether age was associated with differences in outcomes through variation in the type or frequency of strategy use (i.e., whether age differences in strategy use accounted for age differences in outcomes), testing emotion-regulatory strategies as mediators. In contrast, moderation analyses tested whether the associations between strategy use and outcomes differed by age. Finally, studies examining emotional work demands addressed whether exposure to such demands, or their associations with outcomes, differed across age, either by modelling age as a moderator of the relationship between emotional work demands and outcomes or by comparing these associations across age groups. Within each domain, findings are structured based on the analytical role of age. In studies of emotion-regulatory strategies, findings are further organized according to the analytical role of these strategies (e.g., as mediators or moderators) in the examined age–outcome relationships. Given the heterogeneity in study designs, measures, and analytical approaches, the results are presented as a narrative synthesis that prioritizes identifying overarching patterns and inconsistencies across studies. Individual studies are referred to selectively to illustrate these patterns, while detailed study characteristics and results are reported in Table 2.

Across the reviewed studies, findings did not converge on a consistent account of how age, emotion-regulatory strategies, and emotional work demands relate to health and work outcomes. Across studies, differing assumptions were made regarding whether age differences in outcomes reflect variation in the use of specific strategies, differences in how these strategies are associated with outcomes, or age-related differences in how emotional work demands are related to outcomes. However, empirical findings do not consistently support any of these assumptions. Instead, results were mixed and, in some cases, contradictory, with some studies suggesting comparatively more favourable associations for older workers, others indicating reversed patterns, and others finding no age differences. In addition, findings on emotional work demands suggest that age-related effects vary depending on contextual factors, further limiting general conclusions. Overall, it remains unclear whether age-related differences in outcomes are primarily driven by differences in strategy use, differences in how strategies are related to outcomes, or context-specific influences of emotional work demands. Thus, the reviewed evidence reveals neither a consistent pattern of age-related effects nor on a common conceptualization of the role of age in the relationships between emotion-regulatory strategies, emotional work demands, and health and work outcomes.

### Emotion-regulatory strategies

From the 19 studies examining emotion-regulatory strategies, nine studies tested age as a moderator [38, 39, 40–42, 44–46], two studies compared age groups [47, 48], and eight studies tested mediations [4, 49, 50–53–55]. Johnson [51] tested both the mediating and the moderating role of emotional labour strategies, and is therefore reported twice, separately for each assumption test.

### Age as a moderator of associations between emotion-regulatory strategies and outcomes

Most studies examining age as a moderator of associations between emotion-regulatory strategies and health and work outcomes found that the examined strategies were associated with comparatively more favourable outcomes in older than in younger workers, a pattern often interpreted as reflecting age-related advantages in emotion regulation. For example, Peng et al. [39] found that suppression was linked to lower physical strain and higher well-being among older compared to younger employees. Similarly, Yeung and Fung [45] and Yeung and Wong [46] reported that suppression was associated with fewer negative emotions, lower stress, and higher job satisfaction among older workers. Deep acting was associated with lower burnout in Khetjenkarn and Agmapisarn [38] and with higher perceived work ability in Rudolph and McGonagle [41], with these associations being more favourable among older compared to younger employees. In addition, Peng et al. [40] found that cognitive reappraisal was associated with lower organisational deviance among older workers. However, not all findings were consistent with this pattern. Walsh and Bartikowski [44] reported a positive association between deep acting and job satisfaction among younger, but not older employees, and a positive association between surface acting and quitting intentions among older, but not younger workers. Across these studies, age differences were primarily reflected in the strength and direction of associations between strategy use and health and work outcomes, rather than in consistent differences in the frequency or type of strategy use across age groups.

A few studies examined age differences in the antecedents of emotion regulation or in strategy use itself. Scheibe [43] showed that age differences in the activation of emotion-regulation goals were associated with differences in negative affect, pointing to age-related variation in regulatory antecedents rather than in the effectiveness of specific strategies. Similarly, Scheibe and Moghimi [42] found that, in response to more intense negative events, younger employees increased their use of suppression and decreased their use of acceptance more than older employees, patterns that were associated with lower job satisfaction and higher fatigue.

Taken together, these studies suggest that age may moderate the associations between emotion-regulatory strategies and outcomes. However, evidence for such moderation was inconsistent. Observed differences varied in direction across studies, and some studies found no moderating effects. While some findings indicated comparatively more favourable associations for older workers for certain strategies, others showed reversed effects, and it remains unclear whether these differences reflect variation in strategy use or differences in how strategies are associated with outcomes across age groups.

### Age group comparisons in associations between emotion-regulatory strategies and outcomes

The two studies comparing age groups in the associations between emotion-regulatory strategies and outcomes also yielded inconsistent findings regarding age differences. One study suggested that these associations may be stronger among younger than older employees, whereas the other found no age differences. Specifically, Kafetsios and Loumakou [47] found that reappraisal was associated with higher positive job affect and job satisfaction and lower negative job affect among younger employees, whereas these associations were not observed among older employees. In contrast, suppression in the younger group was associated with lower positive job affect and job satisfaction and higher negative job affect. These findings were interpreted as indicating that emotion-regulation strategies may be more strongly related to affective work experiences and job satisfaction among younger compared to older employees, with limited evidence for such associations in older workers. In contrast, Kaur and Malodia [48] reported a negative association between emotional labour and job satisfaction and found no significant age differences in either emotional labour or job satisfaction. Taken together, these findings provide limited and inconsistent evidence regarding age differences in the associations between emotion-regulatory strategies and job affect and job satisfaction, with one study suggesting stronger associations among younger employees and the other providing no evidence of age-related differences.

### Emotion-regulatory strategies as mediators of age–outcome associations

Across the eight studies examining emotion-regulatory strategies as mediators of age–outcome associations, findings were mixed. Five studies reported significant indirect effects linking age to health and work outcomes via emotion-regulatory strategies, whereas three found no support for such mediation. Among studies reporting indirect effects, the specific strategies, outcomes, and model structures varied considerably, and no consistent mediating mechanism emerged.

For example, Cheung and Tang [49] found that deep acting partially mediated the relationship between age and job satisfaction, indicating that higher levels of deep acting among older workers were associated with higher job satisfaction. Johnson et al. [51] also reported multiple indirect effects, with surface acting mediating the relationships between age and lower exhaustion and cynicism, and anticipative deep acting mediating the relationships between age and higher professional efficacy and work engagement. Peng et al. [54] identified a more complex, serial mediation pathway in which surface acting, deep acting, and organizational cynicism jointly mediated the relationship between age and workplace deviance.

In contrast, three studies [50, 53, 55] found no support for emotion-regulatory strategies as mediators of age–outcome associations. In these studies, mediation was not supported because age was not related to the proposed mediators or because the mediators did not significantly contribute to the indirect pathways, challenging the assumption that emotion-regulatory strategies explain age-related differences in work and health outcomes.

### Emotion-regulatory strategies as moderators of age–outcome associations

Evidence for emotion-regulatory strategies as moderators of age–outcome associations was limited. Johnson et al. [51] did not find support for a two-way interaction between age and emotional labour strategies and only identified a three-way interaction. This interaction indicated that the association between age and professional efficacy varied depending on both deep acting and customer-related stressors, with differing patterns under high versus low stress. No additional age-related interaction effects were observed for other strategies or outcomes. These findings provide limited evidence for a moderating role of emotion-regulatory strategies in age–outcome associations, with only a single, context-specific effect identified.

### Emotional work demands

Studies investigating whether emotional work demands interacted with age in predicting health or work outcomes or compared effects across age groups, revealed mixed results. In these studies, age moderation was tested in Mijakoski et al. [56], Reh et al. [57], Scheibe et al. [58], and Van der Heijden et al. [59], while age groups were compared in Irehill et al. [60], Mauno et al. [61], and Zoer et al. [62]. Studies on emotional work demands generally focused on age-related differences in the associations between various types of emotional work demands and health and work outcomes.

### Age as moderator of associations between emotional work demands and outcomes

Findings regarding age as a moderator of associations between emotional work demands and outcomes were mixed and context-dependent. While several studies indicated that emotional work demands altered age–outcome relationships, the direction of these effects varied across contexts and outcomes. For example, Reh et al. [57] found that emotional well-being, reflected in positive affect, decreased with age, whereas job satisfaction remained relatively stable; however, under very high emotional job demands, steeper age-related declines were observed in both outcomes. In contrast, Scheibe et al. [58] reported a more differentiated pattern, in which age-related differences depended on the type of emotional job demand. Specifically, high emotional sensitivity demands were associated with a greater need for recovery among older compared to younger employees, whereas no age-related differences were observed for display demands. Other studies highlighted context-specific patterns. Van der Heijden et al. [59] found that emotional demands predicted burnout among older, but not younger nurses in the Netherlands, whereas the reverse pattern was observed in Poland. Similarly, Mijakoski et al. [56] reported that high emotional demands were associated with higher depersonalization among older nurses in Macedonia, but not among Croatian nurses. Taken together, these findings indicate that the moderating role of age in the relationship between emotional work demands and outcomes varies across contexts and does not follow a consistent age-related pattern.

### Age group comparisons in associations between emotional work demands and outcomes

Across the three studies comparing age groups, findings did not indicate a consistent pattern of age-related advantage or vulnerability in the associations between emotional work demands and outcomes. Instead, effects varied across age groups and outcomes. Specifically, Mauno et al. [61] found that emotional sensitivity demands were more negatively related to job satisfaction and work dedication among middle-aged employees than among younger or older workers, suggesting greater vulnerability in mid-career. Zoer et al. [62] reported that moderate to high emotional workload was associated with increased stress, fatigue, and burnout only among younger workers, whereas work pressure was related to these outcomes across all age groups. In contrast, Irehill et al. [60] found no age differences in the associations between emotional demands and burnout or vigour among leaders. Together, these findings indicate that age differences in the associations between emotional work demands and outcomes are inconsistent and possibly context-dependent.

## Discussion

This review synthesized evidence on how age relates to emotion-regulatory strategies, emotional work demands, and health and work outcomes. Across the reviewed studies, no single, consistent pattern of age-related differences emerged that would suggest a clear advantage or disadvantage for any age group in how emotion-regulatory processes or emotional work demands relate to health and work outcomes. While some findings indicate comparatively more favourable associations between certain emotion-regulatory strategies and outcomes among older workers, other studies show reversed effects or no age differences. Similarly, findings on emotional work demands indicate that the associations between demands and health and work outcomes vary as a function of age, but age-related differences are not consistently observed and, when present, do not follow a clear pattern of advantage or disadvantage. The results do not converge on a coherent explanation of age-related differences in how emotion-regulatory strategies and emotional work demands relate to health and work outcomes. Rather than reflecting a single underlying pattern, the observed heterogeneity may be partially explained by studies testing different, and often implicit assumptions about how age, emotion-regulatory strategies, emotional work demands, and outcomes are related. Some studies conceptualized age-related variation in health and work outcomes as reflecting differences in the frequency or types of emotion-regulatory strategies used. Others examined whether the associations of emotion-regulatory strategies or emotional work demands with health and work outcomes varied as a function of age. In the following, we interpret these patterns in relation to the mechanisms proposed by SST and the SAVI model. These theoretical frameworks are used here as interpretive lenses to contextualize the findings. However, the reviewed studies rarely test the core mechanisms proposed by these frameworks, and the present synthesis does not provide direct support for their explanatory assumptions in workplace contexts. We also consider methodological and contextual factors that may account for the observed heterogeneity.

### Age differences in associations between emotion-regulatory strategies and outcomes

Concerning the first research question, the reviewed studies suggest that age may influence the relationship between emotion-regulatory strategies and health and work outcomes. However, this influence is not consistent. While some findings indicate that certain strategies are associated with comparatively more favourable health or work outcomes among older workers, other studies report reversed patterns or no age-related differences. Overall, the evidence does not support a clear or systematic age-related advantage or disadvantage in how emotion-regulatory strategies relate to outcomes.

This pattern suggests that age may shape the associations between emotion-regulatory strategies and outcomes, but not in a uniform or predictable way. While some findings suggest that emotion-regulatory strategies may contribute to explaining age-related differences in outcomes, this evidence is mixed and not consistently observed. Importantly, the reviewed studies do not allow firm conclusions about whether observed age differences primarily reflect variation in the frequency or types of emotion-regulatory strategies used, or variation in how these strategies are associated with health and work outcomes across age groups. Taken together, these findings suggest that emotion-regulatory strategies may contribute to age-related differences in health and work outcomes. However, the reviewed evidence neither consistently supports this interpretation nor allows firm conclusions about how age and emotion-regulatory strategies jointly influence health and work outcomes.

### Age differences in associations between emotional work demands and outcomes

Concerning the second research question, the reviewed studies suggest that age may influence the relationship between emotional work demands and health and work outcomes; however, this influence is not consistent. Across studies, age-related differences in these associations are sometimes observed, but vary in direction and are not consistently present. Overall, the evidence does not support a clear or systematic pattern of age-related advantages or disadvantages in how emotional work demands influence health and work outcomes.

This variability indicates that the effects of emotional work demands are not determined by age alone, but are likely shaped by contextual factors such as differences in work environments and job roles, which may influence how emotional work demands are experienced and managed. Age therefore appears to act as one of several factors influencing how emotional work demands relate to health and work outcomes, rather than as a primary or consistent determinant. Taken together, these findings suggest that emotional work demands do not affect workers uniformly across age groups, but instead show context-dependent associations, limiting the ability to draw general conclusions about age-related differences.

### Theoretical implications: socioemotional selectivity theory

SST [23, 63] proposes that age-related shifts in perceived time horizons lead to a prioritization of emotionally meaningful goals, which in turn may influence emotional experiences and well-being. Although several reviewed studies interpret age-related differences in emotion-regulatory strategies through this framework, the empirical evidence provides only partial and indirect support for SST in workplace contexts.

On the one hand, some findings have been interpreted as consistent with SST’s proposition that older individuals prioritize emotional well-being, as reflected in comparatively more favourable associations between certain emotion-regulatory strategies or emotional work demands and health or work outcomes among older workers. However, these studies do not directly test SST’s core mechanisms, such as shifts in perceived time horizons or goal priorities, and instead infer such processes from observed age differences in outcomes. On the other hand, these patterns are not consistent and are, in several cases, reversed or absent, which challenges the notion of a general age-related advantage in emotional functioning at work. Moreover, the reviewed studies do not clearly distinguish whether observed differences reflect variation in the frequency of emotion-regulatory strategies used, variation in the types of emotion-regulatory strategies used, or variation in how emotion-regulatory strategies are associated with outcomes across age groups. SST does not explicitly address these distinctions, as its primary focus is on motivational shifts associated with perceived future time horizons, rather than on age-related differences in emotion-regulatory strategy use or in how these strategies are associated with health and work outcomes. This may partly explain why studies drawing on SST have conceptualised the role of age in different ways.

A further limitation concerns the operationalization of age. SST is grounded in perceived future time horizons, yet the reviewed studies predominantly rely on chronological age as a proxy without assessing subjective time perspective. As a result, it remains unclear whether observed age differences reflect SST-consistent motivational processes or other factors such as career stage or work experience.

Finally, SST assumes that individuals increasingly prioritize emotionally meaningful interactions by selectively engaging in rewarding social environments [63]. In organizational settings, however, such selectivity may be constrained, as employees often have limited control over their interaction partners. These structural constraints may limit the applicability of SST’s mechanisms in workplace contexts and help explain why age-related advantages are not consistently observed.

Taken together, while SST provides a useful theoretical framework, the reviewed findings offer only limited support for its application in workplace contexts. In particular, the lack of direct tests of its core mechanisms, the reliance on chronological age, and the constraints of workplace settings limit its ability to account for the variability observed in age-related differences in how emotional work demands and emotion-regulatory strategies are linked to health and work outcomes.

### Strength and vulnerability integration

SAVI [5] proposes that aging is associated with emotional strengths in situations characterised by low or moderate emotional demands, limited duration of exposure, and opportunities for effective emotion regulation, but increased vulnerability under sustained or high-intensity emotional strain. Some findings are consistent with this perspective [4, 57], particularly those indicating that high emotional demands may attenuate otherwise favourable age-related patterns in well-being. However, the evidence is not fully consistent with SAVI’s vulnerability assumption. In some cases, older workers exhibited lower emotional reactivity and more favourable outcomes even under conditions of high emotional strain [43], which does not align with the model’s prediction of increased vulnerability. This suggests that the boundary conditions under which vulnerability emerges in workplace contexts remain insufficiently specified. Overall, while SAVI may offer a useful framework for understanding how emotional work demands and emotion-regulatory processes jointly relate to health and work outcomes, the current evidence provides only partial and inconsistent support and highlights the need for more precise specification of the conditions under which age-related strengths and vulnerabilities emerge.

### Conceptualizing and measuring age in the included studies

Beyond theoretical considerations, the way age is operationalized in the included studies presents challenges. Many studies relied on relatively young samples and used arbitrary thresholds to define “older” workers, often well below the ages at which age-related differences in emotion regulation have been examined in the broader literature (e.g., a meta-analysis comparing younger adults with samples aged 60 years and above [24]). This mismatch raises questions about whether the observed findings can meaningfully speak to age-related differences in emotion-regulatory processes in later adulthood, or if they reflect variation within earlier stages of the working lifespan. Notably, even in studies including adults aged 60 years and above, evidence for age-related differences in emotion regulation remains limited and inconsistent [24]. Thus, the reliance on substantially younger samples in the reviewed studies further constrains the ability to conclude age-related differences in emotional work demands and emotion-regulatory processes at work, and their associations with health and work outcomes.

In addition, age was frequently treated as a linear predictor or dichotomous category, limiting the ability to capture potential non-linear patterns across the working lifespan. This approach may obscure meaningful variation across different life or career stages.

Finally, most studies focused exclusively on chronological age and did not consider other relevant age-related dimensions, such as work experience, organizational tenure, or career stage. In workplace contexts, these factors may more directly shape emotional work demands and the capacity or resources for regulating emotions than age itself. The omission of such variables limits the ability to disentangle effects attributable to aging from those related to professional development or role characteristics.

Taken together, these issues highlight the need for a more multidimensional and theoretically grounded conceptualization of age when examining how age and age-related factors may shape the associations between emotional work demands, emotion-regulatory strategies, and health and work outcomes.

### Potential confounders in the included studies

A key limitation across the reviewed studies is the limited consideration of contextual and individual-level confounders that are likely to shape both emotional work demands and emotion-regulatory processes. Emotional work demands and emotion at work rarely occur in isolation but are embedded in broader organisational and psychosocial contexts, including factors such as workload, autonomy, role clarity, and social support. These conditions may systematically vary across job roles and career stages and can either buffer or exacerbate emotional strain. As such, the absence of these variables in most studies makes it difficult to isolate the specific contribution of emotional work demands or emotion-regulatory strategies to the observed outcomes and may help explain the inconsistent age-related patterns identified in this review.

Another important, yet rarely addressed, source of bias is the healthy worker effect [64, 65], which may imply that older workers who remain in emotionally demanding roles represent a selectively resilient subgroup, whereas those who experience adverse outcomes may exit earlier through role changes or labour market withdrawal. This selection process may lead to an overrepresentation of emotionally resilient older workers in cross-sectional samples, potentially inflating observed age-related differences.

In addition, individual differences in psychological resources, such as emotional intelligence, coping styles, and personality traits, were seldom measured or controlled for, despite their established role in shaping emotional experiences and regulation [21, 66, 67]. The omission of these factors further limits the ability to disentangle age-related differences from individual variability.

Taken together, the limited consideration of contextual, selection, and individual-level factors constrains the interpretation of age-related effects and may contribute to the variability and lack of consistent patterns observed across studies.

### Methodological concerns in the included studies

Beyond the conceptual and contextual limitations discussed above, several additional methodological concerns further constrain the interpretation of age-related differences in emotional work demands and emotion-regulatory strategies. Firstly, most studies relied on cross-sectional or repeated cross-sectional designs, limiting the ability to draw causal inferences or to distinguish age effects from cohort or selection effects. The limited use of longitudinal designs further restricts insight into how emotional work demands and regulation may change across the working lifespan. Second, the heavy reliance on self-report measures introduces potential biases and may inflate associations between variables. Although some studies employed experience sampling methods, these approaches were uncommon. Thus, much of the evidence is based on designs that are not well-suited to capturing within-person variability and the context-dependent nature of emotional work demands and emotion-regulatory processes. Finally, variability in methodological quality and reporting practices may have contributed to the heterogeneity of findings. Study populations were often not theoretically justified, and the selection of measures was not always clearly motivated, limiting transparency and comparability across studies.

### Limitations of the review

Despite a transparent and systematic review process, several limitations remain that may have shaped the inclusion of studies and the interpretation of findings. Although a comprehensive search strategy was employed across multiple databases, some relevant studies were identified only through citation tracking. This suggests that the initial keyword strategy may not have fully captured the breadth of relevant literature. As a result, there is a risk of search bias, particularly for studies using alternative terminology or conceptualizing emotion-regulatory strategies and emotional work demands in less conventional ways. In addition, restricting the review to peer-reviewed published research introduces the possibility of publication bias, as studies reporting null or unexpected results are less likely to appear in published outlets [68]. Although the review included articles reporting any age-related findings, even when age was not the primary focus, to minimise the risk of excluding relevant age-related evidence, some relevant studies may nevertheless have been missed.

The review’s scope and inclusion criteria also introduced certain trade-offs. Studies focusing on individual-level traits, such as emotional intelligence, coping, or conflict management skills, were excluded, as they emphasise personal attributes rather than emotional work demands, or regulatory strategies embedded in the work environment. While this ensured conceptual clarity, it may have excluded emotional competencies that are influenced by age and relevant to workplace functioning. Moreover, the substantial heterogeneity in study designs, age definitions, outcome measures, and conceptualizations of emotional work demands and regulation strategies complicated synthesis. As a result, the interpretation was necessarily more narrative and critical than cumulative. These challenges underscore the need for greater conceptual consistency and methodological rigor in future research on age, emotional work demands, and emotion regulation in occupational settings.

Finally, although methodological quality was assessed using the QuADS tool, findings were not formally weighted according to quality scores. Given the heterogeneity in study designs, constructs, outcomes, and analytical approaches, the appraisal was used descriptively to inform interpretation rather than to stratify findings.

## Conclusions and need for future research

This review synthesised evidence on how age relates to emotion-regulatory strategies, emotional work demands, and health and work outcomes. Across the reviewed studies, no consistent or generalisable pattern of age-related differences emerged. While several studies suggest comparatively more favourable associations between certain emotion-regulatory strategies and outcomes among older compared to younger workers, others show reversed effects or no age differences. Similarly, the associations between emotional work demands and outcomes varied by age, but did not follow a uniform direction. The findings indicate that age did not emerge as a robust or consistent factor in how emotion-regulatory strategies or emotional work demands relate to health and work outcomes. Instead, the observed variability suggests that age-related differences may be context-dependent and shaped by a combination of occupational, situational, and lifespan-related factors. However, the inconsistency of the existing literature appears to reflect not only heterogeneous empirical findings, but also theoretical and conceptual fragmentation. One of the main conceptual contributions of this review is to highlight these sources of inconsistency. First, the included studies rarely explicitly tested the mechanisms proposed by the theories or frameworks they invoked. Second, studies reporting age differences varied in what aspect these differences reflected, including variation in the frequency of emotion-regulatory strategies used, variation in the types of emotion-regulatory strategies used, variation in how these strategies were associated with health and work outcomes across age, and variation in how emotional work demands were associated with health and work outcomes across age. Recognising these sources of theoretical and conceptual fragmentation is important for developing a more coherent conceptual framework to guide future research.

In addition to developing a theoretically sound and conceptually clear framework, future research would benefit from adopting more integrative approaches that explicitly examine how emotional work demands, emotion-regulatory strategies, and health and work outcomes are linked, and where age-related differences may emerge within this process. Addressing potential age-related differences would also require more context-sensitive and methodologically rigorous designs. Longitudinal and within-person approaches could capture how emotional work demands and regulation unfold over time and distinguish age effects from cohort and selection effects. In addition, it would be useful to incorporate relevant contextual and individual-level factors, such as job characteristics, work experience, and individual and work-related resources, to better account for variability in emotional functioning at work. Finally, future research would benefit from defining and justifying age-group comparisons more clearly, including which age groups are expected to differ and why. Expanding research to older segments of the workforce would also be important for understanding whether and how age-related differences emerge in later stages of working life.

From a practical perspective, the findings offer little support for the assumption that older or younger workers are inherently more or less capable of regulating emotions or managing emotional work demands in ways that systematically influence health and work outcomes. Instead, organisations may benefit from recognising that age-related differences in emotional functioning at work may be context-dependent and shaped by organizational, occupational and individual factors beyond chronological age. Consequently, age-inclusive workplace policies and interventions may be more effective when they assess emotional work demands, available resources, and job characteristics directly, rather than tailoring interventions primarily based on workers’ age.

## Supplementary Information

Supplementary Material 1.

Supplementary Material 2.

Supplementary Material 3.

## Data Availability

No new data were generated or analysed in this study. All data supporting the findings are derived from previously published sources, which are cited in the manuscript.
