# Supplementary material for: The influence of age on emotion-regulatory strategies, emotional work demands, and health/work outcomes: a systematic PRISMA literature review with narrative synthesis
Source: BMC Psychol. 2026 Aug 5;14:1136. doi: 10.1186/s40359-026-05277-9 (PMC13439848; doi:10.1186/s40359-026-05277-9)
Supplement: Supplementary file 2 [file 40359_2026_5277_MOESM1_ESM.docx]

**Supplementary material**

*Full search-strings with results*

Updated 2023-09-20

| **Scopus** | | | |
| --- | --- | --- | --- |
| No | Description |  | Items found |
| 1 | Emotional work demands | TITLE-ABS-KEY=  ( "emotion* work*" OR ( emotion* W/2 work* ) OR ( emotion* W/2 regulat* W/2 work* ) OR "emotion* demand*" OR "emotion* labor*" OR "emotion* labour*" ) | 13,616 |
| 2 | Age | TITLE-ABS-KEY=  ( age* OR elder* OR old* OR senior* OR mature* OR junior OR young* OR youth* OR "age difference*" OR lifespan OR "life-span" OR "age-related" OR "age related" ) | 17,719,473 |
| 3 | Specified individual and work-related outcomes | TITLE-ABS-KEY=  ( ( burnout OR burn-out OR "burn out" OR exhaust* OR "emotion* exhaust*" OR fatig* OR depression* OR stress* OR anxiet* OR complain* ) ) OR ( ( mental* OR psych* OR emotion* OR cognit* ) AND ( health* OR wellbeing* OR well-being* OR satisfac* OR symptom* OR ill* OR ail* OR balanc* OR stabil* OR fatig* OR tired* OR abilit* OR capacit* OR vigour* OR vigor* OR energ* OR strengt* OR weak* OR exhaust* ) ) OR ( ( occupation* OR employ* OR work* OR job* OR organisation* OR organization* OR profession* ) AND ( health* OR stress* OR satisfact* OR problem* OR capacit* OR incapacit* OR abilit* OR outcome* OR efficac* OR effectiv* ) ) OR ( ( "recover* W/2 need*" OR turnover OR "turn-over" OR "sick* leave" OR absent* OR productiv* OR perform* ) ) | 23,401,474 |
| 4 | 1 AND 2 | ( TITLE-ABS-KEY ( ( "emotion* work*" OR ( emotion* W/2 work* ) OR ( emotion* W/2 regulat* W/2 work* ) OR "emotion* demand*" OR "emotion* labor*" OR "emotion* labour*" ) ) AND TITLE-ABS-KEY ( ( age* OR elder* OR old* OR senior* OR mature* OR junior OR young* OR youth* OR "age difference*" OR lifespan OR "life-span" OR "age-related" OR "age related" ) ) ) | 3,638 |
| 5 | 1 AND 3 | ( TITLE-ABS-KEY ( ( "emotion* work*" OR ( emotion* W/2 work* ) OR ( emotion* W/2 regulat* W/2 work* ) OR "emotion* demand*" OR "emotion* labor*" OR "emotion* labour*" ) ) AND TITLE-ABS-KEY ( ( ( burnout OR burn-out OR "burn out" OR exhaust* OR "emotion* exhaust*" OR fatig* OR depression* OR stress* OR anxiet* OR complain* ) ) OR ( ( mental* OR psych* OR emotion* OR cognit* ) AND ( health* OR wellbeing* OR well-being* OR satisfac* OR symptom* OR ill* OR ail* OR balanc* OR stabil* OR fatig* OR tired* OR abilit* OR capacit* OR vigour* OR vigor* OR energ* OR strengt* OR weak* OR exhaust* ) ) OR ( ( occupation* OR employ* OR work* OR job* OR organisation* OR organization* OR profession* ) AND ( health* OR stress* OR satisfact* OR problem* OR capacit* OR incapacit* OR abilit* OR outcome* OR efficac* OR effectiv* ) ) OR ( ( "recover* W/2 need*" OR turnover OR "turn-over" OR "sick* leave" OR absent* OR productiv* OR perform* ) ) ) ) | 10,540 |
| 6 | 2 AND 3 | ( TITLE-ABS-KEY ( ( age* OR elder* OR old* OR senior* OR mature* OR junior OR young* OR youth* OR "age difference*" OR lifespan OR "life-span" OR "age-related" OR "age related" ) ) AND TITLE-ABS-KEY ( ( ( burnout OR burn-out OR "burn out" OR exhaust* OR "emotion* exhaust*" OR fatig* OR depression* OR stress* OR anxiet* OR complain* ) ) OR ( ( mental* OR psych* OR emotion* OR cognit* ) AND ( health* OR wellbeing* OR well-being* OR satisfac* OR symptom* OR ill* OR ail* OR balanc* OR stabil* OR fatig* OR tired* OR abilit* OR capacit* OR vigour* OR vigor* OR energ* OR strengt* OR weak* OR exhaust* ) ) OR ( ( occupation* OR employ* OR work* OR job* OR organisation* OR organization* OR profession* ) AND ( health* OR stress* OR satisfact* OR problem* OR capacit* OR incapacit* OR abilit* OR outcome* OR efficac* OR effectiv* ) ) OR ( ( "recover* W/2 need*" OR turnover OR "turn-over" OR "sick* leave" OR absent* OR productiv* OR perform* ) ) ) ) | 5,938,733 |
| 7 | 1 AND 2 AND 3 | ( TITLE-ABS-KEY ( ( "emotion* work*" OR ( emotion* W/2 work* ) OR ( emotion* W/2 regulat* W/2 work* ) OR "emotion* demand*" OR "emotion* labor*" OR "emotion* labour*" ) ) AND TITLE-ABS-KEY ( ( age* OR elder* OR old* OR senior* OR mature* OR junior OR young* OR youth* OR "age difference*" OR lifespan OR "life-span" OR "age-related" OR "age related" ) ) AND TITLE-ABS-KEY ( ( ( burnout OR burn-out OR "burn out" OR exhaust* OR "emotion* exhaust*" OR fatig* OR depression* OR stress* OR anxiet* OR complain* ) ) OR ( ( mental* OR psych* OR emotion* OR cognit* ) AND ( health* OR wellbeing* OR well-being* OR satisfac* OR symptom* OR ill* OR ail* OR balanc* OR stabil* OR fatig* OR tired* OR abilit* OR capacit* OR vigour* OR vigor* OR energ* OR strengt* OR weak* OR exhaust* ) ) OR ( ( occupation* OR employ* OR work* OR job* OR organisation* OR organization* OR profession* ) AND ( health* OR stress* OR satisfact* OR problem* OR capacit* OR incapacit* OR abilit* OR outcome* OR efficac* OR effectiv* ) ) OR ( ( "recover* W/2 need*" OR turnover OR "turn-over" OR "sick* leave" OR absent* OR productiv* OR perform* ) ) ) ) | 3,081 |
| 8 | Filters:  Publication type: article;  Publication stage: final, in press; Language: English,Danish, Norwegian (No available records in Swedish) | ( TITLE-ABS-KEY ( ( "emotion* work*" OR ( emotion* W/2 work* ) OR ( emotion* W/2 regulat* W/2 work* ) OR "emotion* demand*" OR "emotion* labor*" OR "emotion* labour*" ) ) AND TITLE-ABS-KEY ( ( age* OR elder* OR old* OR senior* OR mature* OR junior OR young* OR youth* OR "age difference*" OR lifespan OR "life-span" OR "age-related" OR "age related" ) ) AND TITLE-ABS-KEY ( ( ( burnout OR burn-out OR "burn out" OR exhaust* OR "emotion* exhaust*" OR fatig* OR depression* OR stress* OR anxiet* OR complain* ) ) OR ( ( mental* OR psych* OR emotion* OR cognit* ) AND ( health* OR wellbeing* OR well-being* OR satisfac* OR symptom* OR ill* OR ail* OR balanc* OR stabil* OR fatig* OR tired* OR abilit* OR capacit* OR vigour* OR vigor* OR energ* OR strengt* OR weak* OR exhaust* ) ) OR ( ( occupation* OR employ* OR work* OR job* OR organisation* OR organization* OR profession* ) AND ( health* OR stress* OR satisfact* OR problem* OR capacit* OR incapacit* OR abilit* OR outcome* OR efficac* OR effectiv* ) ) OR ( ( "recover* W/2 need*" OR turnover OR "turn-over" OR "sick* leave" OR absent* OR productiv* OR perform* ) ) ) ) AND ( LIMIT-TO ( PUBSTAGE , "final" ) OR LIMIT-TO ( PUBSTAGE , "aip" ) ) AND ( LIMIT-TO ( DOCTYPE , "ar" ) ) AND ( LIMIT-TO ( LANGUAGE , "English" ) OR LIMIT-TO ( LANGUAGE , "Danish" ) ) | 2,484 |

| **Web of Science** | | | |
| --- | --- | --- | --- |
| No | Description |  | Items found |
| 1 | Emotional work demands | Topic =  ( "emotion* work*" OR ( emotion* NEAR/2 work* ) OR ( emotion* NEAR/2 regulat* NEAR/2 work* ) OR "emotion* demand*" OR "emotion* labor*" OR "emotion* labour*" ) | 11,171 |
| 2 | Age | Topic=  ( age* OR elder* OR old* OR senior* OR mature* OR junior OR young* OR youth* OR "age difference*" OR lifespan OR "life-span" OR "age-related" OR "age related" ) | 8,022,614 |
| 3 | Specified individual and work-related outcomes | Topic=  ( ( burnout OR burn-out OR "burn out" OR exhaust* OR "emotion* exhaust*" OR fatig* OR depression* OR stress* OR anxiet* OR complain* ) ) OR ( ( mental* OR psych* OR emotion* OR cognit* ) AND ( health* OR wellbeing* OR well-being* OR satisfac* OR symptom* OR ill* OR ail* OR balanc* OR stabil* OR fatig* OR tired* OR abilit* OR capacit* OR vigour* OR vigor* OR energ* OR strengt* OR weak* OR exhaust* ) ) OR ( ( occupation* OR employ* OR work* OR job* OR organisation* OR organization* OR profession* ) AND ( health* OR stress* OR satisfact* OR problem* OR capacit* OR incapacit* OR abilit* OR outcome* OR efficac* OR effectiv* ) ) OR ( ( "recover* NEAR/2 need*" OR turnover OR "turn-over" OR "sick* leave" OR absent* OR productiv* OR perform* ) ) | 16,338,383 |
| 4 | 1 AND 2 | ( "emotion* work*" OR ( emotion* NEAR/2 work* ) OR ( emotion* NEAR/2 regulat* NEAR/2 work* ) OR "emotion* demand*" OR "emotion* labor*" OR "emotion* labour*" ) (Topic) and ( age* OR elder* OR old* OR senior* OR mature* OR junior OR young* OR youth* OR "age difference*" OR lifespan OR "life-span" OR "age-related" OR "age related" ) (Topic) | 2,248 |
| 5 | 1 AND 3 | ( "emotion* work*" OR ( emotion* NEAR/2 work* ) OR ( emotion* NEAR/2 regulat* NEAR/2 work* ) OR "emotion* demand*" OR "emotion* labor*" OR "emotion* labour*" ) (Topic) and ( ( burnout OR burn-out OR "burn out" OR exhaust* OR "emotion* exhaust*" OR fatig* OR depression* OR stress* OR anxiet* OR complain* ) ) OR ( ( mental* OR psych* OR emotion* OR cognit* ) AND ( health* OR wellbeing* OR well-being* OR satisfac* OR symptom* OR ill* OR ail* OR balanc* OR stabil* OR fatig* OR tired* OR abilit* OR capacit* OR vigour* OR vigor* OR energ* OR strengt* OR weak* OR exhaust* ) ) OR ( ( occupation* OR employ* OR work* OR job* OR organisation* OR organization* OR profession* ) AND ( health* OR stress* OR satisfact* OR problem* OR capacit* OR incapacit* OR abilit* OR outcome* OR efficac* OR effectiv* ) ) OR ( ( "recover* NEAR/2 need*" OR turnover OR "turn-over" OR "sick* leave" OR absent* OR productiv* OR perform* ) ) (Topic) | 8,857 |
| 6 | 2 AND 3 | ( age* OR elder* OR old* OR senior* OR mature* OR junior OR young* OR youth* OR "age difference*" OR lifespan OR "life-span" OR "age-related" OR "age related" ) (Topic) and ( ( burnout OR burn-out OR "burn out" OR exhaust* OR "emotion* exhaust*" OR fatig* OR depression* OR stress* OR anxiet* OR complain* ) ) OR ( ( mental* OR psych* OR emotion* OR cognit* ) AND ( health* OR wellbeing* OR well-being* OR satisfac* OR symptom* OR ill* OR ail* OR balanc* OR stabil* OR fatig* OR tired* OR abilit* OR capacit* OR vigour* OR vigor* OR energ* OR strengt* OR weak* OR exhaust* ) ) OR ( ( occupation* OR employ* OR work* OR job* OR organisation* OR organization* OR profession* ) AND ( health* OR stress* OR satisfact* OR problem* OR capacit* OR incapacit* OR abilit* OR outcome* OR efficac* OR effectiv* ) ) OR ( ( "recover* NEAR/2 need*" OR turnover OR "turn-over" OR "sick* leave" OR absent* OR productiv* OR perform* ) ) (Topic) | [2,855,060](https://www.webofscience.com/wos/woscc/summary/41e7caa8-2e7c-402a-807b-87405f4d75d7-a532024e/relevance/1) |
| 7 | 1 AND 2 AND 3 | ( "emotion* work*" OR ( emotion* NEAR/2 work* ) OR ( emotion* NEAR/2 regulat* NEAR/2 work* ) OR "emotion* demand*" OR "emotion* labor*" OR "emotion* labour*" ) (Topic) and ( age* OR elder* OR old* OR senior* OR mature* OR junior OR young* OR youth* OR "age difference*" OR lifespan OR "life-span" OR "age-related" OR "age related" ) (Topic) and ( ( burnout OR burn-out OR "burn out" OR exhaust* OR "emotion* exhaust*" OR fatig* OR depression* OR stress* OR anxiet* OR complain* ) ) OR ( ( mental* OR psych* OR emotion* OR cognit* ) AND ( health* OR wellbeing* OR well-being* OR satisfac* OR symptom* OR ill* OR ail* OR balanc* OR stabil* OR fatig* OR tired* OR abilit* OR capacit* OR vigour* OR vigor* OR energ* OR strengt* OR weak* OR exhaust* ) ) OR ( ( occupation* OR employ* OR work* OR job* OR organisation* OR organization* OR profession* ) AND ( health* OR stress* OR satisfact* OR problem* OR capacit* OR incapacit* OR abilit* OR outcome* OR efficac* OR effectiv* ) ) OR ( ( "recover* NEAR/2 need*" OR turnover OR "turn-over" OR "sick* leave" OR absent* OR productiv* OR perform* ) ) (Topic) | 1,880 |
| 8 | Filters:  Publication type: article;  Language: English,Danish, Norwegian (No available reconds in Swedish | ( "emotion* work*" OR ( emotion* NEAR/2 work* ) OR ( emotion* NEAR/2 regulat* NEAR/2 work* ) OR "emotion* demand*" OR "emotion* labor*" OR "emotion* labour*" ) (Topic) and ( age* OR elder* OR old* OR senior* OR mature* OR junior OR young* OR youth* OR "age difference*" OR lifespan OR "life-span" OR "age-related" OR "age related" ) (Topic) and ( ( burnout OR burn-out OR "burn out" OR exhaust* OR "emotion* exhaust*" OR fatig* OR depression* OR stress* OR anxiet* OR complain* ) ) OR ( ( mental* OR psych* OR emotion* OR cognit* ) AND ( health* OR wellbeing* OR well-being* OR satisfac* OR symptom* OR ill* OR ail* OR balanc* OR stabil* OR fatig* OR tired* OR abilit* OR capacit* OR vigour* OR vigor* OR energ* OR strengt* OR weak* OR exhaust* ) ) OR ( ( occupation* OR employ* OR work* OR job* OR organisation* OR organization* OR profession* ) AND ( health* OR stress* OR satisfact* OR problem* OR capacit* OR incapacit* OR abilit* OR outcome* OR efficac* OR effectiv* ) ) OR ( ( "recover* NEAR/2 need*" OR turnover OR "turn-over" OR "sick* leave" OR absent* OR productiv* OR perform* ) ) (Topic) and Articles (Document Types) and English or Danish or Norwegian (Languages) | 1,620 |
| **CINAHL** | | | |
| No | Description |  | Items found |
| 1 | Emotional work demands | TX = all text=  ( "emotion* work*" OR ( emotion* W2 work* ) OR ( emotion* W2 regulat* W2 work* ) OR "emotion* demand*" OR "emotion* labor*" OR "emotion* labour*" ) | 2,315 |
| 2 | Age | TX = all text=  ( age* OR elder* OR old* OR senior* OR mature* OR junior OR young* OR youth* OR "age difference*" OR lifespan OR "life-span" OR "age-related" OR "age related" ) | 2,936,402 |
| 3 | Specified individual and work-related outcomes | TX = all text=  ( ( burnout OR burn-out OR "burn out" OR exhaust* OR "emotion* exhaust*" OR fatig* OR depression* OR stress* OR anxiet* OR complain* ) ) OR ( ( mental* OR psych* OR emotion* OR cognit* ) AND ( health* OR wellbeing* OR well-being* OR satisfac* OR symptom* OR ill* OR ail* OR balanc* OR stabil* OR fatig* OR tired* OR abilit* OR capacit* OR vigour* OR vigor* OR energ* OR strengt* OR weak* OR exhaust* ) ) OR ( ( occupation* OR employ* OR work* OR job* OR organisation* OR organization* OR profession* ) AND ( health* OR stress* OR satisfact* OR problem* OR capacit* OR incapacit* OR abilit* OR outcome* OR efficac* OR effectiv* ) ) OR ( ( "recover* W2 need*" OR turnover OR "turn-over" OR "sick* leave" OR absent* OR productiv* OR perform* ) ) | 3,063,587 |
| 4 | 1 AND 2 | TX ( ( "emotion* work*" OR ( emotion* W2 work* ) OR ( emotion* W2 regulat* W2 work* ) OR "emotion* demand*" OR "emotion* labor*" OR "emotion* labour*" ) ) AND TX ( ( age* OR elder* OR old* OR senior* OR mature* OR junior OR young* OR youth* OR "age difference*" OR lifespan OR "life-span" OR "age-related" OR "age related" ) ) | 996 |
| 5 | 1 AND 3 | TX ( ( "emotion* work*" OR ( emotion* W2 work* ) OR ( emotion* W2 regulat* W2 work* ) OR "emotion* demand*" OR "emotion* labor*" OR "emotion* labour*" ) ) AND TX ( ( ( burnout OR burn-out OR "burn out" OR exhaust* OR "emotion* exhaust*" OR fatig* OR depression* OR stress* OR anxiet* OR complain* ) ) OR ( ( mental* OR psych* OR emotion* OR cognit* ) AND ( health* OR wellbeing* OR well-being* OR satisfac* OR symptom* OR ill* OR ail* OR balanc* OR stabil* OR fatig* OR tired* OR abilit* OR capacit* OR vigour* OR vigor* OR energ* OR strengt* OR weak* OR exhaust* ) ) OR ( ( occupation* OR employ* OR work* OR job* OR organisation* OR organization* OR profession* ) AND ( health* OR stress* OR satisfact* OR problem* OR capacit* OR incapacit* OR abilit* OR outcome* OR efficac* OR effectiv* ) ) OR ( ( "recover* W2 need*" OR turnover OR "turn-over" OR "sick* leave" OR absent* OR productiv* OR perform* ) ) ) | 2183 |
| 6 | 2 AND 3 | TX ( ( age* OR elder* OR old* OR senior* OR mature* OR junior OR young* OR youth* OR "age difference*" OR lifespan OR "life-span" OR "age-related" OR "age related" ) ) AND TX ( ( ( burnout OR burn-out OR "burn out" OR exhaust* OR "emotion* exhaust*" OR fatig* OR depression* OR stress* OR anxiet* OR complain* ) ) OR ( ( mental* OR psych* OR emotion* OR cognit* ) AND ( health* OR wellbeing* OR well-being* OR satisfac* OR symptom* OR ill* OR ail* OR balanc* OR stabil* OR fatig* OR tired* OR abilit* OR capacit* OR vigour* OR vigor* OR energ* OR strengt* OR weak* OR exhaust* ) ) OR ( ( occupation* OR employ* OR work* OR job* OR organisation* OR organization* OR profession* ) AND ( health* OR stress* OR satisfact* OR problem* OR capacit* OR incapacit* OR abilit* OR outcome* OR efficac* OR effectiv* ) ) OR ( ( "recover* W2 need*" OR turnover OR "turn-over" OR "sick* leave" OR absent* OR productiv* OR perform* ) ) ) | 1,382,839 |
| 7 | 1 AND 2 AND 3 | TX ( ( "emotion* work*" OR ( emotion* W2 work* ) OR ( emotion* W2 regulat* W2 work* ) OR "emotion* demand*" OR "emotion* labor*" OR "emotion* labour*" ) ) AND TX ( ( age* OR elder* OR old* OR senior* OR mature* OR junior OR young* OR youth* OR "age difference*" OR lifespan OR "life-span" OR "age-related" OR "age related" ) ) AND TX ( ( ( burnout OR burn-out OR "burn out" OR exhaust* OR "emotion* exhaust*" OR fatig* OR depression* OR stress* OR anxiet* OR complain* ) ) OR ( ( mental* OR psych* OR emotion* OR cognit* ) AND ( health* OR wellbeing* OR well-being* OR satisfac* OR symptom* OR ill* OR ail* OR balanc* OR stabil* OR fatig* OR tired* OR abilit* OR capacit* OR vigour* OR vigor* OR energ* OR strengt* OR weak* OR exhaust* ) ) OR ( ( occupation* OR employ* OR work* OR job* OR organisation* OR organization* OR profession* ) AND ( health* OR stress* OR satisfact* OR problem* OR capacit* OR incapacit* OR abilit* OR outcome* OR efficac* OR effectiv* ) ) OR ( ( "recover* W2 need*" OR turnover OR "turn-over" OR "sick* leave" OR absent* OR productiv* OR perform* ) ) ) | 955 |
| 8 | Filters activated: Academic journal; Peer reviewed, Language: English (Swedish, Danish, Norwegian are not available) |  | 833 |

| **PsycInfo** | | | |
| --- | --- | --- | --- |
| No | Description |  | Items found |
| 1 | Emotional work demands | TX= all text=  ( "emotion* work*" OR ( emotion* W2 work* ) OR ( emotion* W2 regulat* W2 work* ) OR "emotion* demand*" OR "emotion* labor*") | 5,430 |
| 2 | Age | TX = all text=  ( age* OR elder* OR old* OR senior* OR mature* OR junior OR young* OR youth* OR "age difference*" OR lifespan OR "life-span" OR "age-related" OR "age related" ) | 3,176,572 |
| 3 | Specified individual and work-related outcomes | TX = all text=  ( ( burnout OR burn-out OR "burn out" OR exhaust* OR "emotion* exhaust*" OR fatig* OR depression* OR stress* OR anxiet* OR complain* ) ) OR ( ( mental* OR psych* OR emotion* OR cognit* ) AND ( health* OR wellbeing* OR well-being* OR satisfac* OR symptom* OR ill* OR ail* OR balanc* OR stabil* OR fatig* OR tired* OR abilit* OR capacit* OR vigour* OR vigor* OR energ* OR strengt* OR weak* OR exhaust* ) ) OR ( ( occupation* OR employ* OR work* OR job* OR organisation* OR organization* OR profession* ) AND ( health* OR stress* OR satisfact* OR problem* OR capacit* OR incapacit* OR abilit* OR outcome* OR efficac* OR effectiv* ) ) OR ( ( "recover* W2 need*" OR turnover OR "turn-over" OR "sick* leave" OR absent* OR productiv* OR perform* ) ) | 3,357,842 |
| 4 | 1 AND 2 | TX ( ( "emotion* work*" OR ( emotion* W2 work* ) OR ( emotion* W2 regulat* W2 work* ) OR "emotion* demand*" OR "emotion* labor*") ) AND TX ( ( age* OR elder* OR old* OR senior* OR mature* OR junior OR young* OR youth* OR "age difference*" OR lifespan OR "life-span" OR "age-related" OR "age related" ) ) | 4,112 |
| 5 | 1 AND 3 | TX ( ( "emotion* work*" OR ( emotion* W2 work* ) OR ( emotion* W2 regulat* W2 work* ) OR "emotion* demand*" OR "emotion* labor*") ) AND TX ( ( ( burnout OR burn-out OR "burn out" OR exhaust* OR "emotion* exhaust*" OR fatig* OR depression* OR stress* OR anxiet* OR complain* ) ) OR ( ( mental* OR psych* OR emotion* OR cognit* ) AND ( health* OR wellbeing* OR well-being* OR satisfac* OR symptom* OR ill* OR ail* OR balanc* OR stabil* OR fatig* OR tired* OR abilit* OR capacit* OR vigour* OR vigor* OR energ* OR strengt* OR weak* OR exhaust* ) ) OR ( ( occupation* OR employ* OR work* OR job* OR organisation* OR organization* OR profession* ) AND ( health* OR stress* OR satisfact* OR problem* OR capacit* OR incapacit* OR abilit* OR outcome* OR efficac* OR effectiv* ) ) OR ( ( "recover* W2 need*" OR turnover OR "turn-over" OR "sick* leave" OR absent* OR productiv* OR perform* ) ) ) | 4,698 |
| 6 | 2 AND 3 | TX ( ( age* OR elder* OR old* OR senior* OR mature* OR junior OR young* OR youth* OR "age difference*" OR lifespan OR "life-span" OR "age-related" OR "age related" ) ) AND TX ( ( ( burnout OR burn-out OR "burn out" OR exhaust* OR "emotion* exhaust*" OR fatig* OR depression* OR stress* OR anxiet* OR complain* ) ) OR ( ( mental* OR psych* OR emotion* OR cognit* ) AND ( health* OR wellbeing* OR well-being* OR satisfac* OR symptom* OR ill* OR ail* OR balanc* OR stabil* OR fatig* OR tired* OR abilit* OR capacit* OR vigour* OR vigor* OR energ* OR strengt* OR weak* OR exhaust* ) ) OR ( ( occupation* OR employ* OR work* OR job* OR organisation* OR organization* OR profession* ) AND ( health* OR stress* OR satisfact* OR problem* OR capacit* OR incapacit* OR abilit* OR outcome* OR efficac* OR effectiv* ) ) OR ( ( "recover* W2 need*" OR turnover OR "turn-over" OR "sick* leave" OR absent* OR productiv* OR perform* ) ) ) | 2,163,478 |
| 7 | 1 AND 2 AND 3 | TX ( ( "emotion* work*" OR ( emotion* W2 work* ) OR ( emotion* W2 regulat* W2 work* ) OR "emotion* demand*" OR "emotion* labor*") ) AND TX ( ( age* OR elder* OR old* OR senior* OR mature* OR junior OR young* OR youth* OR "age difference*" OR lifespan OR "life-span" OR "age-related" OR "age related" ) ) AND TX ( ( ( burnout OR burn-out OR "burn out" OR exhaust* OR "emotion* exhaust*" OR fatig* OR depression* OR stress* OR anxiet* OR complain* ) ) OR ( ( mental* OR psych* OR emotion* OR cognit* ) AND ( health* OR wellbeing* OR well-being* OR satisfac* OR symptom* OR ill* OR ail* OR balanc* OR stabil* OR fatig* OR tired* OR abilit* OR capacit* OR vigour* OR vigor* OR energ* OR strengt* OR weak* OR exhaust* ) ) OR ( ( occupation* OR employ* OR work* OR job* OR organisation* OR organization* OR profession* ) AND ( health* OR stress* OR satisfact* OR problem* OR capacit* OR incapacit* OR abilit* OR outcome* OR efficac* OR effectiv* ) ) OR ( ( "recover* W2 need*" OR turnover OR "turn-over" OR "sick* leave" OR absent* OR productiv* OR perform* ) ) ) | 3,642 |
| 8 | Filters activated: Academic journal; Peer reviewed, Language: English (Swedish, Danish, Norwegian are not available) |  | 2,722 |

| **PuBMed** | | | |
| --- | --- | --- | --- |
| No | Description |  | Items found |
| 1 | Emotional work demands | “emotion work”[title/abstract:~2] OR “emotions work” [title/abstract:~2] OR “emotional work” [title/abstract:~2] OR “emotion works” [title/abstract:~2] OR “emotion working” [title/abstract:~2] OR “emotions works” [title/abstract:~2] OR “emotions working” [title/abstract:~2] OR “emotional works” [title/abstract:~2] OR “emotional working” [title/abstract:~2] OR “emotion regulation work” [title/abstract:~2] OR “emotions regulation work” [title/abstract:~2] OR “emotional regulation work” [title/abstract:~2] OR “emotion regulations work” [title/abstract:~2] OR “emotions regulations work” [title/abstract:~2] OR “emotional regulations work” [title/abstract:~2] OR “emotion regulation works” [title/abstract:~2] OR “emotion regulation working” [title/abstract:~2] OR “emotions regulation works” [title/abstract:~2] OR “emotions regulations working” [title/abstract:~2] OR “emotional regulation works” [title/abstract:~2] OR “emotional regulation working” [title/abstract:~2] OR “emotion regulations works” [title/abstract:~2] OR “emotion regulations working” [title/abstract:~2] OR “emotions regulations works” [title/abstract:~2] OR “emotions regulations working” [title/abstract:~2] OR “emotional regulations works” [title/abstract:~2] OR “emotional regulations working” [title/abstract:~2] OR "emotion demand*"[title/abstract] OR “emotions demand*”[title/abstract] OR “emotional demand*”[title/abstract] OR "emotion labor*"[title/abstract] OR “emotions labor*”[title/abstract] OR “emotional labor*”[title/abstract] | 3,266 |
| 2 | Age | (age[title/abstract] OR aged[title/abstract] OR ageing[title/abstract] OR aging[title/abstract] OR elder*[title/abstract] OR old[title/abstract] OR older[title/abstract] OR oldest[title/abstract] OR senior*[title/abstract] OR mature*[title/abstract] OR junior[title/abstract] OR young*[title/abstract] OR youth*[title/abstract] OR "age difference*"[title/abstract] OR lifespan[title/abstract] OR "life-span"[title/abstract] OR "age-related"[title/abstract] OR "age related"[title/abstract]) | [5,258,184](https://pubmed.ncbi.nlm.nih.gov/?term=%28age%5Btitle%2Fabstract%5D+OR+aged%5Btitle%2Fabstract%5D+OR+ageing%5Btitle%2Fabstract%5D+OR+aging%5Btitle%2Fabstract%5D+OR+elder%2A%5Btitle%2Fabstract%5D+OR+old%5Btitle%2Fabstract%5D+OR+older%5Btitle%2Fabstract%5D+OR+oldest%5Btitle%2Fabstract%5D+OR+senior%2A%5Btitle%2Fabstract%5D+OR+mature%2A%5Btitle%2Fabstract%5D+OR+junior%5Btitle%2Fabstract%5D+OR+young%2A%5Btitle%2Fabstract%5D+OR+youth%2A%5Btitle%2Fabstract%5D+OR+%22age+difference%2A%22%5Btitle%2Fabstract%5D+OR+lifespan%5Btitle%2Fabstract%5D+OR+%22life-span%22%5Btitle%2Fabstract%5D+OR+%22age-related%22%5Btitle%2Fabstract%5D+OR+%22age+related%22%5Btitle%2Fabstract%5D%29&sort=) |
| 3 | Specified individual and work-related outcomes | ( ( burnout[title/abstract] OR burn-out[title/abstract] OR "burn out"[title/abstract] OR exhaust*[title/abstract] OR "emotion exhaust*"[title/abstract] OR "emotions exhaust*"[title/abstract] OR "emotional exhaust*"[title/abstract] OR fatig*[title/abstract] OR depression*[title/abstract] OR stress*[title/abstract] OR anxiet*[title/abstract] OR complain*[title/abstract]) ) OR ( ( mental*[title/abstract] OR psych*[title/abstract] OR emotion*[title/abstract] OR cognit*[title/abstract]) AND ( health*[title/abstract] OR wellbeing*[title/abstract] OR well-being*[title/abstract] OR satisfac*[title/abstract] OR symptom*[title/abstract] OR ill[title/abstract] OR illness[title/abstract] OR ail[title/abstract] OR ailment[title/abstract] OR balanc*[title/abstract] OR stabil*[title/abstract] OR fatig*[title/abstract] OR tired*[title/abstract] OR abilit*[title/abstract] OR capacit*[title/abstract] OR vigour*[title/abstract] OR vigor*[title/abstract] OR energ*[title/abstract] OR strengt*[title/abstract] OR weak* [title/abstract] OR exhaust*[title/abstract]) ) OR ( ( occupation*[title/abstract] OR employ*[title/abstract] OR work*[title/abstract] OR job[title/abstract] OR jobs[title/abstract] OR organisation*[title/abstract] OR organization*[title/abstract] OR profession*[title/abstract]) AND ( health*[title/abstract] OR stress*[title/abstract] OR satisfact*[title/abstract] OR problem*[title/abstract] OR capacit*[title/abstract] OR incapacit*[title/abstract] OR abilit*[title/abstract] OR outcome*[title/abstract] OR efficac*[title/abstract] OR effectiv*[title/abstract]) ) OR ( ( turnover[title/abstract] OR "turn-over"[title/abstract] OR absent*[title/abstract] OR productiv*[title/abstract] OR perform*[title/abstract]) ) | 7,790,733 |
| 4 | 1 AND 2 | **("emotion work"[title/abstract:~2] OR "emotions work" [title/abstract:~2] OR "emotional work" [title/abstract:~2] OR "emotion works" [title/abstract:~2] OR "emotion working" [title/abstract:~2] OR "emotions works" [title/abstract:~2] OR "emotions working" [title/abstract:~2] OR "emotional works" [title/abstract:~2] OR "emotional working" [title/abstract:~2] OR "emotion regulation work" [title/abstract:~2] OR "emotions regulation work" [title/abstract:~2] OR "emotional regulation work" [title/abstract:~2] OR "emotion regulations work" [title/abstract:~2] OR "emotions regulations work" [title/abstract:~2] OR "emotional regulations work" [title/abstract:~2] OR "emotion regulation works" [title/abstract:~2] OR "emotion regulation working" [title/abstract:~2] OR "emotions regulation works" [title/abstract:~2] OR "emotions regulations working" [title/abstract:~2] OR "emotional regulation works" [title/abstract:~2] OR "emotional regulation working" [title/abstract:~2] OR "emotion regulations works" [title/abstract:~2] OR "emotion regulations working" [title/abstract:~2] OR "emotions regulations works" [title/abstract:~2] OR "emotions regulations working" [title/abstract:~2] OR "emotional regulations works" [title/abstract:~2] OR "emotional regulations working" [title/abstract:~2] OR "emotion demand*"[title/abstract] OR "emotions demand*"[title/abstract] OR "emotional demand*"[title/abstract] OR "emotion labor*"[title/abstract] OR "emotions labor*"[title/abstract] OR "emotional labor*"[title/abstract]) AND ((age[title/abstract] OR aged[title/abstract] OR ageing[title/abstract] OR aging[title/abstract] OR elder*[title/abstract] OR old[title/abstract] OR older[title/abstract] OR oldest[title/abstract] OR senior*[title/abstract] OR mature*[title/abstract] OR junior[title/abstract] OR young*[title/abstract] OR youth*[title/abstract] OR "age difference*"[title/abstract] OR lifespan[title/abstract] OR "life-span"[title/abstract] OR "age-related"[title/abstract] OR "age related"[title/abstract]))** | 812 |
| 5 | 1 AND 3 | **("emotion work"[title/abstract:~2] OR "emotions work" [title/abstract:~2] OR "emotional work" [title/abstract:~2] OR "emotion works" [title/abstract:~2] OR "emotion working" [title/abstract:~2] OR "emotions works" [title/abstract:~2] OR "emotions working" [title/abstract:~2] OR "emotional works" [title/abstract:~2] OR "emotional working" [title/abstract:~2] OR "emotion regulation work" [title/abstract:~2] OR "emotions regulation work" [title/abstract:~2] OR "emotional regulation work" [title/abstract:~2] OR "emotion regulations work" [title/abstract:~2] OR "emotions regulations work" [title/abstract:~2] OR "emotional regulations work" [title/abstract:~2] OR "emotion regulation works" [title/abstract:~2] OR "emotion regulation working" [title/abstract:~2] OR "emotions regulation works" [title/abstract:~2] OR "emotions regulations working" [title/abstract:~2] OR "emotional regulation works" [title/abstract:~2] OR "emotional regulation working" [title/abstract:~2] OR "emotion regulations works" [title/abstract:~2] OR "emotion regulations working" [title/abstract:~2] OR "emotions regulations works" [title/abstract:~2] OR "emotions regulations working" [title/abstract:~2] OR "emotional regulations works" [title/abstract:~2] OR "emotional regulations working" [title/abstract:~2] OR "emotion demand*"[title/abstract] OR "emotions demand*"[title/abstract] OR "emotional demand*"[title/abstract] OR "emotion labor*"[title/abstract] OR "emotions labor*"[title/abstract] OR "emotional labor*"[title/abstract]) AND (( ( burnout[title/abstract] OR burn-out[title/abstract] OR "burn out"[title/abstract] OR exhaust*[title/abstract] OR "emotion exhaust*"[title/abstract] OR "emotions exhaust*"[title/abstract] OR "emotional exhaust*"[title/abstract] OR fatig*[title/abstract] OR depression*[title/abstract] OR stress*[title/abstract] OR anxiet*[title/abstract] OR complain*[title/abstract]) ) OR ( ( mental*[title/abstract] OR psych*[title/abstract] OR emotion*[title/abstract] OR cognit*[title/abstract]) AND ( health*[title/abstract] OR wellbeing*[title/abstract] OR well-being*[title/abstract] OR satisfac*[title/abstract] OR symptom*[title/abstract] OR ill[title/abstract] OR illness[title/abstract] OR ail[title/abstract] OR ailment[title/abstract] OR balanc*[title/abstract] OR stabil*[title/abstract] OR fatig*[title/abstract] OR tired*[title/abstract] OR abilit*[title/abstract] OR capacit*[title/abstract] OR vigour*[title/abstract] OR vigor*[title/abstract] OR energ*[title/abstract] OR strengt*[title/abstract] OR weak* [title/abstract] OR exhaust*[title/abstract]) ) OR ( ( occupation*[title/abstract] OR employ*[title/abstract] OR work*[title/abstract] OR job[title/abstract] OR jobs[title/abstract] OR organisation*[title/abstract] OR organization*[title/abstract] OR profession*[title/abstract]) AND ( health*[title/abstract] OR stress*[title/abstract] OR satisfact*[title/abstract] OR problem*[title/abstract] OR capacit*[title/abstract] OR incapacit*[title/abstract] OR abilit*[title/abstract] OR outcome*[title/abstract] OR efficac*[title/abstract] OR effectiv*[title/abstract]) ) OR ( ( turnover[title/abstract] OR "turn-over"[title/abstract] OR absent*[title/abstract] OR productiv*[title/abstract] OR perform*[title/abstract]) ))** | 2,858 |
| 6 | 2 AND 3 | **((age[title/abstract] OR aged[title/abstract] OR ageing[title/abstract] OR aging[title/abstract] OR elder*[title/abstract] OR old[title/abstract] OR older[title/abstract] OR oldest[title/abstract] OR senior*[title/abstract] OR mature*[title/abstract] OR junior[title/abstract] OR young*[title/abstract] OR youth*[title/abstract] OR "age difference*"[title/abstract] OR lifespan[title/abstract] OR "life-span"[title/abstract] OR "age-related"[title/abstract] OR "age related"[title/abstract])) AND (( ( burnout[title/abstract] OR burn-out[title/abstract] OR "burn out"[title/abstract] OR exhaust*[title/abstract] OR "emotion exhaust*"[title/abstract] OR "emotions exhaust*"[title/abstract] OR "emotional exhaust*"[title/abstract] OR fatig*[title/abstract] OR depression*[title/abstract] OR stress*[title/abstract] OR anxiet*[title/abstract] OR complain*[title/abstract]) ) OR ( ( mental*[title/abstract] OR psych*[title/abstract] OR emotion*[title/abstract] OR cognit*[title/abstract]) AND ( health*[title/abstract] OR wellbeing*[title/abstract] OR well-being*[title/abstract] OR satisfac*[title/abstract] OR symptom*[title/abstract] OR ill[title/abstract] OR illness[title/abstract] OR ail[title/abstract] OR ailment[title/abstract] OR balanc*[title/abstract] OR stabil*[title/abstract] OR fatig*[title/abstract] OR tired*[title/abstract] OR abilit*[title/abstract] OR capacit*[title/abstract] OR vigour*[title/abstract] OR vigor*[title/abstract] OR energ*[title/abstract] OR strengt*[title/abstract] OR weak* [title/abstract] OR exhaust*[title/abstract]) ) OR ( ( occupation*[title/abstract] OR employ*[title/abstract] OR work*[title/abstract] OR job[title/abstract] OR jobs[title/abstract] OR organisation*[title/abstract] OR organization*[title/abstract] OR profession*[title/abstract]) AND ( health*[title/abstract] OR stress*[title/abstract] OR satisfact*[title/abstract] OR problem*[title/abstract] OR capacit*[title/abstract] OR incapacit*[title/abstract] OR abilit*[title/abstract] OR outcome*[title/abstract] OR efficac*[title/abstract] OR effectiv*[title/abstract]) ) OR ( ( turnover[title/abstract] OR "turn-over"[title/abstract] OR absent*[title/abstract] OR productiv*[title/abstract] OR perform*[title/abstract]) ))** | 1,901,253 |
| 7 | 1 AND 2 AND 3 | **(("emotion work"[title/abstract:~2] OR "emotions work" [title/abstract:~2] OR "emotional work" [title/abstract:~2] OR "emotion works" [title/abstract:~2] OR "emotion working" [title/abstract:~2] OR "emotions works" [title/abstract:~2] OR "emotions working" [title/abstract:~2] OR "emotional works" [title/abstract:~2] OR "emotional working" [title/abstract:~2] OR "emotion regulation work" [title/abstract:~2] OR "emotions regulation work" [title/abstract:~2] OR "emotional regulation work" [title/abstract:~2] OR "emotion regulations work" [title/abstract:~2] OR "emotions regulations work" [title/abstract:~2] OR "emotional regulations work" [title/abstract:~2] OR "emotion regulation works" [title/abstract:~2] OR "emotion regulation working" [title/abstract:~2] OR "emotions regulation works" [title/abstract:~2] OR "emotions regulations working" [title/abstract:~2] OR "emotional regulation works" [title/abstract:~2] OR "emotional regulation working" [title/abstract:~2] OR "emotion regulations works" [title/abstract:~2] OR "emotion regulations working" [title/abstract:~2] OR "emotions regulations works" [title/abstract:~2] OR "emotions regulations working" [title/abstract:~2] OR "emotional regulations works" [title/abstract:~2] OR "emotional regulations working" [title/abstract:~2] OR "emotion demand*"[title/abstract] OR "emotions demand*"[title/abstract] OR "emotional demand*"[title/abstract] OR "emotion labor*"[title/abstract] OR "emotions labor*"[title/abstract] OR "emotional labor*"[title/abstract]) AND ((age[title/abstract] OR aged[title/abstract] OR ageing[title/abstract] OR aging[title/abstract] OR elder*[title/abstract] OR old[title/abstract] OR older[title/abstract] OR oldest[title/abstract] OR senior*[title/abstract] OR mature*[title/abstract] OR junior[title/abstract] OR young*[title/abstract] OR youth*[title/abstract] OR "age difference*"[title/abstract] OR lifespan[title/abstract] OR "life-span"[title/abstract] OR "age-related"[title/abstract] OR "age related"[title/abstract]))) AND (( ( burnout[title/abstract] OR burn-out[title/abstract] OR "burn out"[title/abstract] OR exhaust*[title/abstract] OR "emotion exhaust*"[title/abstract] OR "emotions exhaust*"[title/abstract] OR "emotional exhaust*"[title/abstract] OR fatig*[title/abstract] OR depression*[title/abstract] OR stress*[title/abstract] OR anxiet*[title/abstract] OR complain*[title/abstract]) ) OR ( ( mental*[title/abstract] OR psych*[title/abstract] OR emotion*[title/abstract] OR cognit*[title/abstract]) AND ( health*[title/abstract] OR wellbeing*[title/abstract] OR well-being*[title/abstract] OR satisfac*[title/abstract] OR symptom*[title/abstract] OR ill[title/abstract] OR illness[title/abstract] OR ail[title/abstract] OR ailment[title/abstract] OR balanc*[title/abstract] OR stabil*[title/abstract] OR fatig*[title/abstract] OR tired*[title/abstract] OR abilit*[title/abstract] OR capacit*[title/abstract] OR vigour*[title/abstract] OR vigor*[title/abstract] OR energ*[title/abstract] OR strengt*[title/abstract] OR weak* [title/abstract] OR exhaust*[title/abstract]) ) OR ( ( occupation*[title/abstract] OR employ*[title/abstract] OR work*[title/abstract] OR job[title/abstract] OR jobs[title/abstract] OR organisation*[title/abstract] OR organization*[title/abstract] OR profession*[title/abstract]) AND ( health*[title/abstract] OR stress*[title/abstract] OR satisfact*[title/abstract] OR problem*[title/abstract] OR capacit*[title/abstract] OR incapacit*[title/abstract] OR abilit*[title/abstract] OR outcome*[title/abstract] OR efficac*[title/abstract] OR effectiv*[title/abstract]) ) OR ( ( turnover[title/abstract] OR "turn-over"[title/abstract] OR absent*[title/abstract] OR productiv*[title/abstract] OR perform*[title/abstract]) ))** | 731 |
| 8 | Filters activated: Language: English,Swedish, Danish, Norwegian | **(("emotion work"[title/abstract:~2] OR "emotions work" [title/abstract:~2] OR "emotional work" [title/abstract:~2] OR "emotion works" [title/abstract:~2] OR "emotion working" [title/abstract:~2] OR "emotions works" [title/abstract:~2] OR "emotions working" [title/abstract:~2] OR "emotional works" [title/abstract:~2] OR "emotional working" [title/abstract:~2] OR "emotion regulation work" [title/abstract:~2] OR "emotions regulation work" [title/abstract:~2] OR "emotional regulation work" [title/abstract:~2] OR "emotion regulations work" [title/abstract:~2] OR "emotions regulations work" [title/abstract:~2] OR "emotional regulations work" [title/abstract:~2] OR "emotion regulation works" [title/abstract:~2] OR "emotion regulation working" [title/abstract:~2] OR "emotions regulation works" [title/abstract:~2] OR "emotions regulations working" [title/abstract:~2] OR "emotional regulation works" [title/abstract:~2] OR "emotional regulation working" [title/abstract:~2] OR "emotion regulations works" [title/abstract:~2] OR "emotion regulations working" [title/abstract:~2] OR "emotions regulations works" [title/abstract:~2] OR "emotions regulations working" [title/abstract:~2] OR "emotional regulations works" [title/abstract:~2] OR "emotional regulations working" [title/abstract:~2] OR "emotion demand*"[title/abstract] OR "emotions demand*"[title/abstract] OR "emotional demand*"[title/abstract] OR "emotion labor*"[title/abstract] OR "emotions labor*"[title/abstract] OR "emotional labor*"[title/abstract]) AND ((age[title/abstract] OR aged[title/abstract] OR ageing[title/abstract] OR aging[title/abstract] OR elder*[title/abstract] OR old[title/abstract] OR older[title/abstract] OR oldest[title/abstract] OR senior*[title/abstract] OR mature*[title/abstract] OR junior[title/abstract] OR young*[title/abstract] OR youth*[title/abstract] OR "age difference*"[title/abstract] OR lifespan[title/abstract] OR "life-span"[title/abstract] OR "age-related"[title/abstract] OR "age related"[title/abstract]))) AND (( ( burnout[title/abstract] OR burn-out[title/abstract] OR "burn out"[title/abstract] OR exhaust*[title/abstract] OR "emotion exhaust*"[title/abstract] OR "emotions exhaust*"[title/abstract] OR "emotional exhaust*"[title/abstract] OR fatig*[title/abstract] OR depression*[title/abstract] OR stress*[title/abstract] OR anxiet*[title/abstract] OR complain*[title/abstract]) ) OR ( ( mental*[title/abstract] OR psych*[title/abstract] OR emotion*[title/abstract] OR cognit*[title/abstract]) AND ( health*[title/abstract] OR wellbeing*[title/abstract] OR well-being*[title/abstract] OR satisfac*[title/abstract] OR symptom*[title/abstract] OR ill[title/abstract] OR illness[title/abstract] OR ail[title/abstract] OR ailment[title/abstract] OR balanc*[title/abstract] OR stabil*[title/abstract] OR fatig*[title/abstract] OR tired*[title/abstract] OR abilit*[title/abstract] OR capacit*[title/abstract] OR vigour*[title/abstract] OR vigor*[title/abstract] OR energ*[title/abstract] OR strengt*[title/abstract] OR weak* [title/abstract] OR exhaust*[title/abstract]) ) OR ( ( occupation*[title/abstract] OR employ*[title/abstract] OR work*[title/abstract] OR job[title/abstract] OR jobs[title/abstract] OR organisation*[title/abstract] OR organization*[title/abstract] OR profession*[title/abstract]) AND ( health*[title/abstract] OR stress*[title/abstract] OR satisfact*[title/abstract] OR problem*[title/abstract] OR capacit*[title/abstract] OR incapacit*[title/abstract] OR abilit*[title/abstract] OR outcome*[title/abstract] OR efficac*[title/abstract] OR effectiv*[title/abstract]) ) OR ( ( turnover[title/abstract] OR "turn-over"[title/abstract] OR absent*[title/abstract] OR productiv*[title/abstract] OR perform*[title/abstract]) )) Filters: Danish, English, Norwegian, Swedish** | 689 |
