# Supplementary material for: The influence of age on emotion-regulatory strategies, emotional work demands, and health/work outcomes: a systematic PRISMA literature review with narrative synthesis
Source: BMC Psychol. 2026 Aug 5;14:1136. doi: 10.1186/s40359-026-05277-9 (PMC13439848; doi:10.1186/s40359-026-05277-9)
Supplement: Supplementary file 3 [file 40359_2026_5277_MOESM3_ESM.docx]

| **Section and Topic** | **Item #** | **Checklist item** | **Location where item is reported** |
| --- | --- | --- | --- |
| **TITLE** | | |  |
| Title | 1 | Identify the report as a systematic review. | Page 1 |
| **ABSTRACT** | | |  |
| Abstract | 2 | See the PRISMA 2020 for Abstracts checklist. | Pages 2-3 |
| **INTRODUCTION** | | |  |
| Rationale | 3 | Describe the rationale for the review in the context of existing knowledge. | Pages 3-9 |
| Objectives | 4 | Provide an explicit statement of the objective(s) or question(s) the review addresses. | Page 9 |
| **METHODS** | | |  |
| Eligibility criteria | 5 | Specify the inclusion and exclusion criteria for the review and how studies were grouped for the syntheses. | Pages 11-13 |
| Information sources | 6 | Specify all databases, registers, websites, organisations, reference lists and other sources searched or consulted to identify studies. Specify the date when each source was last searched or consulted. | Pages 10-11 |
| Search strategy | 7 | Present the full search strategies for all databases, registers and websites, including any filters and limits used. | Pages 10-11; Supplementary Material |
| Selection process | 8 | Specify the methods used to decide whether a study met the inclusion criteria of the review, including how many reviewers screened each record and each report retrieved, whether they worked independently, and if applicable, details of automation tools used in the process. | Pages 11-12 |
| Data collection process | 9 | Specify the methods used to collect data from reports, including how many reviewers collected data from each report, whether they worked independently, any processes for obtaining or confirming data from study investigators, and if applicable, details of automation tools used in the process. | Page 14 |
| Data items | 10a | List and define all outcomes for which data were sought. Specify whether all results that were compatible with each outcome domain in each study were sought (e.g. for all measures, time points, analyses), and if not, the methods used to decide which results to collect. | Pages 10-11; Page 14 |
|  | 10b | List and define all other variables for which data were sought (e.g. participant and intervention characteristics, funding sources). Describe any assumptions made about any missing or unclear information. | Page 14 |
| Study risk of bias assessment | 11 | Specify the methods used to assess risk of bias in the included studies, including details of the tool(s) used, how many reviewers assessed each study and whether they worked independently, and if applicable, details of automation tools used in the process. | Pages 14-15 |
| Effect measures | 12 | Specify for each outcome the effect measure(s) (e.g. risk ratio, mean difference) used in the synthesis or presentation of results. | Pages 15-16 |
| Synthesis methods | 13a | Describe the processes used to decide which studies were eligible for each synthesis (e.g. tabulating the study intervention characteristics and comparing against the planned groups for each synthesis (item #5)). | Page 15; Pages 18-19 |
|  | 13b | Describe any methods required to prepare the data for presentation or synthesis, such as handling of missing summary statistics, or data conversions. | N. a., no data conversion was required |
|  | 13c | Describe any methods used to tabulate or visually display results of individual studies and syntheses. | Page 15; Pages 1-9 in Tables and figures section |
|  | 13d | Describe any methods used to synthesize results and provide a rationale for the choice(s). If meta-analysis was performed, describe the model(s), method(s) to identify the presence and extent of statistical heterogeneity, and software package(s) used. | Page 15 |
|  | 13e | Describe any methods used to explore possible causes of heterogeneity among study results (e.g. subgroup analysis, meta-regression). | Page 15 |
|  | 13f | Describe any sensitivity analyses conducted to assess robustness of the synthesized results. | N. a., no formal sensitivity analyses conducted |
| Reporting bias assessment | 14 | Describe any methods used to assess risk of bias due to missing results in a synthesis (arising from reporting biases). | Page 16 |
| Certainty assessment | 15 | Describe any methods used to assess certainty (or confidence) in the body of evidence for an outcome. | Page 16 |
| **RESULTS** | | |  |
| Study selection | 16a | Describe the results of the search and selection process, from the number of records identified in the search to the number of studies included in the review, ideally using a flow diagram. | Page 11.  Page 1 in the Tables and Figures section |
|  | 16b | Cite studies that might appear to meet the inclusion criteria, but which were excluded, and explain why they were excluded. | Pages 13-14 |
| Study characteristics | 17 | Cite each included study and present its characteristics. | Page 17:  Pages 2-9 in the Tables and Figures section |
| Risk of bias in studies | 18 | Present assessments of risk of bias for each included study. | Pages 17-18 |
| Results of individual studies | 19 | For all outcomes, present, for each study: (a) summary statistics for each group (where appropriate) and (b) an effect estimate and its precision (e.g. confidence/credible interval), ideally using structured tables or plots. | Pages 18-25, narrative results  Pages 2-9 in the Tables and Figures section |
| Results of syntheses | 20a | For each synthesis, briefly summarise the characteristics and risk of bias among contributing studies. | Pages 18-20 |
|  | 20b | Present results of all statistical syntheses conducted. If meta-analysis was done, present for each the summary estimate and its precision (e.g. confidence/credible interval) and measures of statistical heterogeneity. If comparing groups, describe the direction of the effect. | N.a., narrative synthesis only. |
|  | 20c | Present results of all investigations of possible causes of heterogeneity among study results. | N.a., no formal statistical investigation of heterogeneity |
|  | 20d | Present results of all sensitivity analyses conducted to assess the robustness of the synthesized results. | N.a., no formal sensitivity analyses were conducted |
| Reporting biases | 21 | Present assessments of risk of bias due to missing results (arising from reporting biases) for each synthesis assessed. | N.a., no formal assessment of reporting bias was conducted |
| Certainty of evidence | 22 | Present assessments of certainty (or confidence) in the body of evidence for each outcome assessed. | N.a., No formal certainty assessment conducted |
| **DISCUSSION** | | |  |
| Discussion | 23a | Provide a general interpretation of the results in the context of other evidence. | Pages 26-31 |
|  | 23b | Discuss any limitations of the evidence included in the review. | Pages 28-33 |
|  | 23c | Discuss any limitations of the review processes used. | Pages 33-34 |
|  | 23d | Discuss implications of the results for practice, policy, and future research. | Pages 35-36 |
| **OTHER INFORMATION** | | |  |
| Registration and protocol | 24a | Provide registration information for the review, including register name and registration number, or state that the review was not registered. | Page 3; Page 10 |
|  | 24b | Indicate where the review protocol can be accessed, or state that a protocol was not prepared. | Page 3; Page 10 |
|  | 24c | Describe and explain any amendments to information provided at registration or in the protocol. | Page 16 |
| Support | 25 | Describe sources of financial or non-financial support for the review, and the role of the funders or sponsors in the review. | Page 36 |
| Competing interests | 26 | Declare any competing interests of review authors. | Page 36 |
| Availability of data, code and other materials | 27 | Report which of the following are publicly available and where they can be found: template data collection forms; data extracted from included studies; data used for all analyses; analytic code; any other materials used in the review. | No new datasets were generated for this review. The review is based on previously published studies. Extracted data and analytic materials are not publicly available.. |

*From:*  Page MJ, McKenzie JE, Bossuyt PM, Boutron I, Hoffmann TC, Mulrow CD, et al. The PRISMA 2020 statement: an updated guideline for reporting systematic reviews. BMJ 2021;372:n71. doi: 10.1136/bmj.n71. This work is licensed under CC BY 4.0. To view a copy of this license, visit <https://creativecommons.org/licenses/by/4.0/>
